# A simple function for full‐subsets multiple regression in ecology with R

**DOI:** 10.1002/ece3.4134

**Published:** 2018-05-20

**Authors:** Rebecca Fisher, Shaun K. Wilson, Tsai M. Sin, Ai C. Lee, Tim J. Langlois

**Affiliations:** ^1^ Australian Institute of Marine Science UWA Oceans Institute Crawley WA Australia; ^2^ The UWA Oceans Institute and School of Biological Sciences The University of Western Australia Crawley WA Australia; ^3^ Marine Science Program Department of Parks and Wildlife Kensington WA Australia; ^4^ Tropical Marine Science Institute National University of Singapore Singapore Singapore

**Keywords:** collinearity, complete‐subsets modeling, gam, generalized additive models, information theoretic approaches, multimodel inference, multiple regression

## Abstract

Full‐subsets information theoretic approaches are becoming an increasingly popular tool for exploring predictive power and variable importance where a wide range of candidate predictors are being considered. Here, we describe a simple function in the statistical programming language R that can be used to construct, fit, and compare a complete model set of possible ecological or environmental predictors, given a response variable of interest and a starting generalized additive (mixed) model fit. Main advantages include not requiring a complete model to be fit as the starting point for candidate model set construction (meaning that a greater number of predictors can potentially be explored than might be available through functions such as dredge); model sets that include interactions between factors and continuous nonlinear predictors; and automatic removal of models with correlated predictors (based on a user defined criterion for exclusion). The function takes continuous predictors, which are fitted using smoothers via either gam, gamm (mgcv) or gamm4, as well as factor variables which are included on their own or as two‐level interaction terms within the gam smooth (via use of the “by” argument), or with themselves. The function allows any model to be constructed and used as a null model, and takes a range of arguments that allow control over the model set being constructed, including specifying cyclic and linear continuous predictors, specification of the smoothing algorithm used, and the maximum complexity allowed for smooth terms. The use of the function is demonstrated via case studies that highlight how appropriate model sets can be easily constructed and the broader utility of the approach for exploratory ecology.

## 1 INTRODUCTION

The objectives of field ecology are often to gain insights into the important ecological and environmental drivers of the study system. In many complex ecological systems, there is considerable uncertainty in what variables are most important to include as possible predictors, and ecologists often end up collecting a broad range of environmental (e.g., temperature, light) and ecological (e.g., habitat variables, competitors, predators) candidates. Even where there is clear knowledge that a given environmental variable is important, such as temperature, light, and aragonite saturation in the case of corals (Kleypas, McManus, & Menez, 1999), there may still be uncertainty in how the variables should be summarized for use in ecological models. For example, is it the maximum temperatures, skewness, or even their kurtosis that is important (Ateweberhan & McClanahan, 2010; McClanahan, Ateweberhan, Muhando, Maina, & Mohammed, 2007)? Indeed, considerable insight into the processes involved in driving ecological patterns may be gleaned by considering the predictive power of different summary metrics (Steel et al., 2012).

Inevitably, collinearity among explanatory variables occurs, causing problems with their concurrent use in statistical models (Graham, 2003; Whittingham, Stephens, Bradbury, & Freckleton, 2006). Strategies for dealing with correlated predictor variables include data reduction techniques (such as PCA) to create a reduced set of orthogonal variables or using variance inflation metrics to select a subset of noncollinear variables to include. While data reduction techniques definitively remove collinearity, it is often difficult to disentangle the independent effects and predictive strength of the correlated input variables, potentially confounding interpretation (Freckleton, 2011; Graham, 2003), and/or masking possible cause–effect relationships with the individual input variables. Furthermore, when a PCA axis is found to be an important ecological driver, future studies wishing to measure this must collect a large range of variables, many of which may be functionally irrelevant. Likewise, the often arbitrary choice of which correlated variables are the most “important” and retained means that potentially interesting relationships may be overlooked.

Full‐subsets information theoretic approaches (Burnham & Anderson, 2002) provide a useful alternative to stepwise regression (Mundry & Nunn, 2009) that can alleviate some of the issues with multicollinearity. The general idea of information theoretic approaches is to construct a complete model set and compare all the models in this set using model selection criterion such as Akaike information criterion (AIC), AIC corrected for small sample sizes (AIC_c_, Hurvich & Tsai, 1989), or Bayesian information criterion (Wit, van Heuvel, & Romeijn, 2012). While information theoretic approaches can be used to establish a “best” or most “parsimonious” model (if one exists) using model weights, they are more transparent than traditional backwards selection approaches, allowing all good candidate models to be identified and compared. Where several candidate models have substantial weight, information theoretic approaches allow model averaging and multimodel inference, such that predictions properly account for model uncertainty. By considering all variables in all possible (sensible) combinations, the relative importance of different variables can be properly explored (by summing model weights for each variable, see Burnham & Anderson, 2002) without the risk of inadvertent exclusion of important variables, as can happen with backwise selection.

Several R (R Core Team 2017) packages have been available for some time, such as regsubsets from package leaps (Lumley & Miller, 2009) which fits a complete set of linear models, along with MuMIn (Barton, 2013) and AICcmodavg (Mazerolle, 2016), which have made multimodel inference and model averaging approaches highly accessible to practicing ecologists. The function dredge in the package MuMIn constructs a complete model set based on a fit of the most “complex” model (similar to regsubsets), allows random effects to be included via a mixed modeling framework (lme, lmer, Bates, Mächler, Bolker, & Walker, 2006; Bates, Maechler, Bolker, & Walker, 2015; Pinheiro, Bates, DebRoy, & Sarkar, 2013), and nonlinear relationships through the use of generalized additive models (fit via packages mgcv and gamm4, Wood & Scheipl, 2016; Wood, 2017). Here, we expand the toolkit available to ecologists for fitting full‐subsets multiple regression approaches by providing a simple function in R (https://github.com/beckyfisher/FSSgam) that can be used to construct, fit, and compare a candidate model set based on a range of ecological or environmental predictors. The main advantages of this function over existing packages are as follows: (1) It does not require a complete model to be fit as the starting point for candidate model set construction, meaning that a greater number of predictors can potentially be explored than might be available through functions such as dredge; (2) the function properly handles interactions between factor predictors and continuous “smoothed” predictors through the use of “by” arguments in the call to “s” in gam(m) as well as smooth‐smooth interactions via the use of bivariate calls to “te”; and (3) the function automatically removes models containing correlated predictors from the candidate model set, based on a user‐defined criterion for exclusion. As many ecological processes are inherently nonlinear, our function is based on generalized additive (mixed) models via the mgcv (Wood 2006) and gamm4 (Wood & Scheipl, 2016) packages in R, providing a convenient means of exploring complete model sets for a range of continuous, potentially nonlinear, predictors without the need to define the exact functional form of the relationships between the predictors and the response. The function takes continuous predictors, which are fitted using smoothers via either gam, gamm (mgcv), or gamm4, as well as factor variables which are included on their own or as two‐level interaction terms within the gam smooth (via use of the “by” argument), or with themselves.

## 2 FULL‐SUBSETS FUNCTION

### 2.1 Function inputs

The function has three arguments that must be provided, including a data.frame (use.dat, Appendix S1) containing all variables to include in the analysis; an updatable fitted “test” model fit (test.fit, Appendix S1) generated by a call to gam, gamm (mgcv), or uGamm (MuMIn) using the response variable to be analyzed, specifying any relevant random effects and the family; and a character vector specifying which continuous predictor variables from use.dat to include in the model set (pred.vars.cont, Appendix S1). There are 15 other arguments that control various aspects of the model set constructed and final output, and these are described in detail in Appendix S1, along with their default values.

### 2.2 Model set construction

The function generates a complete model set, based on the pred.vars.cont character vector and/or a second vector specifying those that should be included as factors if required (pred.vars.fact, Appendix S1). Three further arguments control the complexity of the candidate model set constructed, including: whether the model set should include interactions between factor predictors or only their main effects (factor.factor.interactions); whether the model set should include interactions between smooths and factor variables (including factor interactions) as “by” arguments (factor.smooth.interactions); whether the model set should include interactions between smooths and other smooths (smooth.smooth.interactions); and the maximum number of predictors to include in a single model (max.predictors, Appendix S1). Including factor–factor and factor–smooth interactions can dramatically increase the number of models in the candidate set and is not recommended when there are factors with many levels. The function allows control over which factor variables should be included as interactions with each other and with smoothed continuous predictors, as well as which smoothed continuous predictors should be included as interactions with each other (see Appendix S1 for details).

Once all the full‐subsets model elements are defined a complete model set is generated from this combined vector using a repeated call to the R function combn, with the argument m (number of elements to choose) set as 1 (only single variable models) through to the defined maximum number of predictors (max.predictors, Appendix S1). This complete model set is then reduced based on a series of checks that remove models where (1) the total number of included predictors is larger than the user‐specified maximum number of predictors (this can occur when factor interactions are included); (2) factor variables included as “by” arguments in smooths do not also include the factor as a main effect; (3) continuous predictors occur as smooths containing a “by” argument as well as a single predictor; (4) continuous predictors occurring as smooths in a bivariate “te” call as well as a single predictor; and (5) estimated pairwise correlations between any two predictors are too high (cor.cutoff, Appendix S1, defaulting to >0.28 in line with recommendations of Graham 2003).

### 2.3 Model fitting

Once the final model set is constructed, it is converted into a list of model formulae, with all continuous predictors specified as smooth terms via s (with or without a “by” argument, depending on the specific model in the set), with k and “bs=“ defaulting to 5 and “cr,” respectively (k and bs.arg, Appendix S1), with the exception of any that are specified as cyclic (cyclic.vars, Appendix S1), for which bs is set to “cc,” or linear (linear.vars, Appendix S1), which are included as parametric linear predictors. Any terms specified as being part of the null model (null.terms, Appendix S1) are also added during model formula construction.

The foreach function from the package doParallel is used to fit each model in the formula list via a call to update, using the test.fit model supplied by the user, allowing parallel processing if specified (parallel, Appendix S1). Use of update means that all details regarding the choice of gamm (mgcv) or gamm4, family, random structures, and correlation structures can be controlled by the user through the test.fit call and are not modified by the full subsets function.

### 2.4 Function outputs

The full.subsets.gam function returns a named list with six elements, including a data.frame (mod.data.out, Appendix S2) that contains the statistics associated with each model fit; the final used data.frame (used.data, Appendix S2), the matrix of estimated predictor correlations (predictor.correlations, Appendix S2), a list containing the try‐error catch associated with models that failed to fit (failed.models, Appendix S2), a complete list of all successfully fitted models (success.models, Appendix S2), and a list containing variable importance scores for each included predictor (variable.importance, Appendix S2). The mod.data.out table includes AIC_c_ and BIC, delta values (e.g., AIC_c_‐min(AIC_c_)), corresponding weight (ω_*i*_) values (Burnham & Anderson, 2002), an estimate of the model *R* ^2^, and a column indicating the presence of each included predictor variable. Calculating *R* ^2^ values is nontrivial for mixed models (Nakagawa & Schielzeth, 2013), and especially for non‐Gaussian cases and the function allows a range of methods for estimating *R* ^2^ to be specified (r2.type, Appendix S1).

Ideally, the list of failed models should be empty, but when this is not the case interrogating failed.models can be useful for troubleshooting, allowing users to examine which models are not fitting and explore the underlying cause by fitting failed models outside the full.subsets.gam call. When a large number of models fail to fit properly it usually indicates poor specification of the initial test.fit or other arguments in the call to full.subsets.gam, such as the inclusion of factor interactions when there are few data within each factor or that inappropriate variables are being included in the model set. The list of successfully fitted models can be used for multimodel inference and generating model averaged predictions.

## 3 APPLYING THE FULL‐SUBSETS APPROACH

### 3.1 Case study 1: The relative influence of habitat and management on reef fishes

Coral reef fish are highly diverse assemblages that provide important ecosystem services for millions of people (Pratchett, Hoey, & Wilson, 2014). These services are, however, threatened by overfishing (MacNeil et al., 2015; Newton, Côté, Pilling, Jennings, & Dulvy, 2007) and a loss of habitat, in particular corals (Wilson, Graham, & Pratchett, 2006) and the structure they provide (Rogers, Blanchard, & Mumby, 2014). No‐take reserves (NTR) promote higher abundance and biomass of fish (McClanahan, Graham, Wilson, Letourneur, & Fisher, 2009; Russ, 2002) and conserve ecosystem function (Graham et al., 2011). It is clear that NTR cannot prevent large‐scale disturbances, such as heat stress that causes extensive coral loss and decline in fish (Graham et al., 2008; Jones, McCormick, Srinivasan, & Eagle, 2004); however, a reduction in local pressures in NTR may facilitate greater resilience of coral reefs (Hughes, Graham, Jackson, Mumby, & Steneck, 2010). By examining patch reefs of differing habitat quality inside and outside of NTR within the Ningaloo marine park, Wilson et al. (2012) explored how habitat degradation and fishing influenced the abundance and biomass of fish from different functional groups.

**Figure 1:**
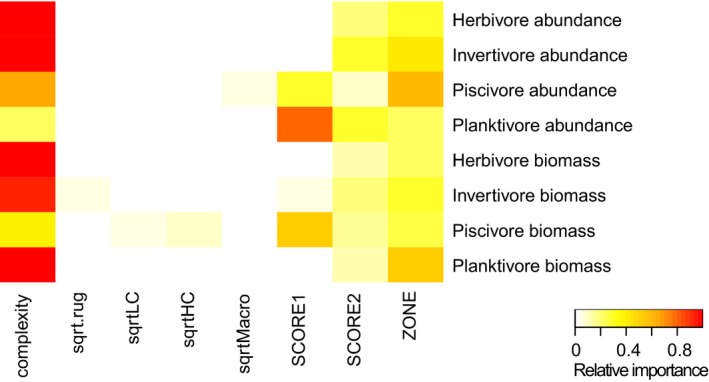
Variable importance scores from a full‐subsets analyses exploring the influence of habitat variables and management zoning on the abundance and biomass of four functional fish feeding guilds (see Appendix S3). Habitat variables included a visual assessment of complexity (complexity); the square root of rugosity (sqrt.rug), cover of low complexity (sqrtLC), and high complexity (sqrtHC) corals and macroalgae cover (sqrtMacro); the first and second axis scores from a principle components analysis (SCORE1 and SCORE2); and management zone (ZONE)

Explanatory variables in the original analyses of Wilson et al., (2007) were summarized using the scores from the two axes of a principal components analysis (PCA), making it impossible to tease apart the relative importance of variables that were correlated along the axis. We re‐analyzed their original data using a full‐subsets multiple regression approach (see details of methods in Appendix S3, along with links to the R code used). The re‐analysis shows clearly that seascape measures of patch reef complexity were generally the best predictor of both fish abundance and biomass (Figure 1, Table A3.1 in Appendix S3). Fish abundance and biomass were low on reefs with no relief and were higher on structurally complex reefs (Figure A3.2 in Appendix S3), a finding consistent with other studies showing that measures of seascape complexity are positively correlated with fish abundance, often outperforming other measures of complexity (Collins et al., 2016; Wilson et al., 2006). The results support the original finding of strong relationships with habitat and only weak evidence for an effect of zoning status on fish abundance and biomass. However, the new analysis teases apart the relative influence of correlated habitat variables, showing that herbivore abundance is strongly influenced by habitat complexity rather than macroalgae. This finding is consistent with other recent studies at Ningaloo that also found abundance of herbivorous fishes is closely related to reef structure rather than macroalgae (Downie, Babcock, Thomson, & Vanderklift, 2013; Wilson et al., 2014). Interestingly, the abundance of planktivores was still strongly related to PCA scores (Figure 1; Figure A3.2 in Appendix S3), suggesting aggregate metrics of habitat may be relevant to some components of the fish assemblage.

### 3.2 Case study 2: The role of reef‐associated predators in structuring adjacent soft‐sediment fauna

Marine no‐take reserves (NTRs) can provide a large‐scale experimental framework for exploring the role of large reef‐associated predators in structuring adjacent soft‐sediment communities (Babcock, Kelly, Shears, Walker, & Willis, 2013; Shears & Babcock, 2002). However, a problem with studies of established NTRs is that evidence based on a negative relationship between predator and prey densities (Hurlbert, 1984; Underwood, Chapman, & Connell, 2000) may be confounded by other covariates also influencing the structure of the soft‐sediment community (e.g., wave action, sediment grain‐size distributions, organic matter, infaunal interactions). Using a dataset collected in northeastern New Zealand, Langlois, Anderson, and Babcock (2005) explored the hypotheses that (1) predation by large reef‐associated predators (sparid fish *Pagrus auratus* and the rock lobster *Jasus edwardsii*) would result in lower densities of large (>4 mm) soft‐sediment macrofauna inside reserves compared to outside reserves (predator model) and (2) predation would decrease with increasing distances from the reef (distance model).

**Figure 2:**
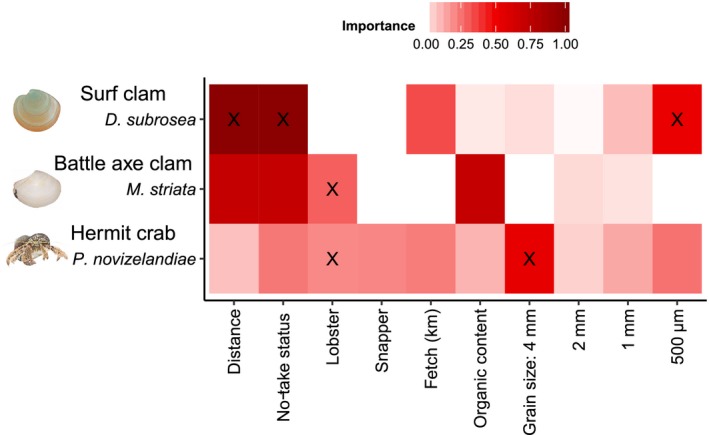
Variable importance scores from full‐subsets analyses of the abundance of *Dosinia subrosea*,* Myadora striata,* and *Pagurus novizelandiae*, with variables within the most parsimonious model for each taxon indicated (X, see Table A4.2 in Appendix S4)

In the original study of Langlois et al. (2005), the influence of environmental variables on the assemblage inside and outside the NTR was investigated using multivariate multiple regression, which found no evidence they were confounding the comparison. Effects on individual taxa were therefore subsequently examined independently using a mixed‐model permutational ANOVA. We revised this original analysis using the full‐subsets multiple regression approach so that the relative importance of NTR status (predator model), distance from the reef edge (distance model), and a range of environmental covariates could be simultaneously evaluated (see details of methods in Appendix S4, along with links to the R code used). We found that the importance of distance from reef and NTR status matched the results of the original study for the bivalve *Dosinia subrosea*. Subsequent manipulative studies have found that *D. subrosea* are readily preyed upon by the large‐bodied rock lobster in the field (Langlois, Anderson, Babcock, & Kato, 2006) and laboratory (Langlois, Anderson, Brock, & Murman, 2006), supporting the results of this analysis. For *Myadora striata* NTR status, distance and organic content were found to be important across all possible models, but a simple model of decreasing abundance of *M. striata* with increasing density of legal‐sized rock lobster was the most parsimonious (Figure 2), corroborating the observation that greater than legal‐size rock lobster can readily prey upon bivalves (Langlois, Anderson, Brock et al., 2006).

There was a high level of model uncertainty in our full‐subsets analysis of the ubiquitous hermit crab *Pagurus novizelandiae*, with very low model weights (maximum weight 0.05, Appendix S4: Table A4.2) and low, relatively evenly distributed variable importance scores (Figure 2). This is consistent with the original study that found no effect of NTR status on the abundance of the ubiquitous hermit crab *Pagurus novizelandiae*. The best model includes the 4‐mm sediment grain‐size fraction and the density of legal‐sized rock lobster (Figure 2). The direct relationship between the density of legal‐sized rock lobster and the hermit crab *P. novizelandiae* supports studies indicating rock lobster can exhibit a strong preference for decapod prey (Dumas, Langlois, Clarke, & Waddington, 2013).

### 3.3 Case study 3: Reproductive cycles of broadcast spawning gastropods over multiple temporal scales

Studies of reproductive biology are fundamental to understanding resource allocation, larval recruitment, and population dynamics (Underwood & Keough, 2001), providing valuable insights into life history strategies, uncovering important interactions with environmental conditions and habitats, and supporting the development of appropriate measures for conservation and management. In addition, an understanding of reproductive cycles and patterns is critical for population modeling and prediction, underpinning efforts to ensure sustainable fishing of commercial species. Reproductive cycles can occur at a number of scales, ranging (in decreasing frequency) from circadian, half‐lunar, and lunar to seasonal. Lunar and semilunar cycles are obvious cues for reproduction, particularly for the broadcast spawners prevalent in marine systems for which synchronicity is critical for fertilization success (Babcock, Mundy, Keesing, & Oliver, 2016). Few studies have concurrently examined effects of annual and lunar patterns on the spawning of marine invertebrates in the tropics in a manner that elucidates relative reproductive output at both temporal scales, with most studies focusing on the annual spawning period. Failure to explicitly model within seasonal patterns can confound data related to reproductive biorhythmicity, as sampling timing often becomes a confounding factor. Part of the issue may relate to the fact that both lunar and seasonal patterns are cyclical in nature, representing a challenge in conventional/traditional analyses.

Here, we take advantage of cyclic general additive models (GAMs) and full‐subsets modeling methods to elucidate reproductive patterns (as indicated by gonadosomatic index, GSI) at multiple temporal scales: (1) among years, (2) among months (i.e., yearly pattern), and (3) within month (i.e., lunar and semilunar cycle) in two species of broadcast spawning gastropods (*Patelloida saccharina* and *Monodonta labio*). The full‐subsets gam function allows other factors (e.g., sex and species) to be examined as both interactions (e.g., a different relationship with lunar day within each level), and as main effects (i.e., a shift in the overall relationship up or down within each level [see details of methods in Appendix S5, along with links to the R code used]). In addition, interactions among continuous smooths can also be explored. Our full‐subsets analysis found that a model with lunar date and month as interactions with species, along with an intercept effect of sex, showed the highest ranking according to both AIC_c_ and BIC, explaining 28% of the variance in GSI for these species (see Table A5.1 in Appendix S5).

**Figure 3:**
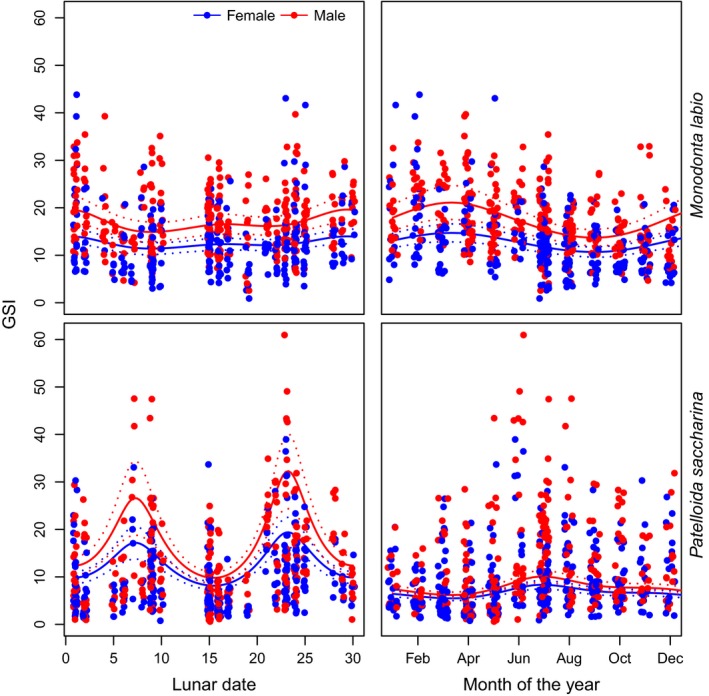
Gonadosomatic index (GSI) as a function of lunar date (left‐hand plots) and month of the year (right‐hand plots) for *Monodonta labio* (upper) and *Patelloida saccharina* (lower), with colors indicating sex (male and female). Fitted gam curves (solid lines) and 95% confidence bands (dashed lines) are also shown

Strong interactions between species and both lunar day and month were due to markedly different trends in GSI for each of these predictors across the two species. A strong semilunar pattern was evident for *P. saccharina*, with fairly equal peaks in GSI occurring around lunar days 7 and 23 and minima occurring around days 0 and 15 (Figure 3). Lunar patterns were generally weak for *M. labio* and also reversed to *P. saccharina*, with peaks centered on lunar days 0 and 15, and minima occurring around days 5 and 23 (Figure 3). Both *P. saccharina* and *M. labio* are continuous breeders, with GSI values remaining above five throughout the year (a phenomenon previously reported in trochids and acmaeids, see Hickman, 1992 for review) (Catalan & Yamamoto, 1993; Creese, 1980). However, there were differences in the timing of peak reproduction between the two species throughout the year, with *M. labio* showing the highest output during February and March, and *P. sacharina* showing peak output in July (Figure 3). February/March denotes the end of the northeast monsoon, with increasing seawater temperatures over the following months, reaching annual peaks around August (Sin et al., 2016). Values of GSI were higher in males compared to females for both species, regardless of lunar day, or time of year (Figure 3), which is a common phenomenon in intertidal gastropods (Creese, 1980; Creese & Ballantine, 1983; Liu, 1994).

## 4 DISCUSSION

The case studies presented clearly demonstrate the value of the full‐subsets information theoretic approach. Our function allows exploration of a wide range of (even correlated) predictors that can elucidate important underlying functional relationships. This is a clear alternative to restrictive and oftentimes flawed null‐hypothesis testing approaches, even in the case of relatively small sets of candidate predictors. Overall, the results of the revised analysis of the relative importance of habitat and management on fish abundance and biomass were generally similar to those from the original study based on PCA. However, by including the underlying habitat information, the new analysis provides a clearer picture of which elements of the benthic assemblage are most important to fish. This additional information is useful for building scientific hypotheses and parametric models for how such fishes may be influenced by changes in habitat (such as is expected due to ocean warming and climate change, Pratchett, Wilson, & Munday, 2015), as well as informing which elements of the habitat should be a focus for management targets. In the benthic infauna case study, the full‐subsets approach allowed alternative hypotheses regarding predator influence and environmental factors to be explicitly disentangled, instead of relying on a simple comparison of NTR status. The flexibility provided by the full‐subsets function is evident from the case study exploring reproduction in broadcast spawning gastropods, showing how complex interactions between factors (species, sex) and multiple temporal scales of periodicity can be thoroughly explored. Although the model set for the gastropod reproduction case study was relatively small (only 52 models in the final set), manually coding all 52 models would be tedious and the inclusion of only a few additional environmental covariates (such as temperature) would render manual formulation of the model set intractable.

The full‐subsets function was developed primarily with the aim of fitting appropriate model sets using generalized additive mixed (GAM) modeling methods with “by” arguments supplied in the smooth call. We focus on the use of GAM here because many ecological processes are inherently nonlinear. While parametric relationships have advantages over GAM approaches as they provide parameterization of the functional relationships between the predictors and the response, defining the exact functional form of these relationships can be quite tedious, particularly in a full‐subsets multiple regression framework. Smoothing methods, such as those implemented via gam, gamm (mgcv) and gamm4, provide a convenient means of exploring the relative predictive power and importance of a range of continuous predictors, given an optimal smoothed relationship. In our GAM fits, we restrict the “*k*” parameter (the dimension of the basis used to represent the smooth term) to reduce overfitting and to ensure largely monotonic relationships. Highly complex functional relationships requiring high *k* values are probably not well suited to full‐subsets multiple regression approach, as partial relationships for models containing multiple continuous predictors can become difficult to interpret.

While functionally similar to dredge, the approach of building the model set from the “bottom up” along with automatic removal of models containing correlated predictors provides subtle yet important differences in the types of model sets that can be easily constructed. Furthermore, our function overcomes issues associated with inclusion of factor variable interactions as “by” arguments in GAM(M) which introduces complexities that are not well handled by dredge. By “hard coding” factor interactions, they can be included as interactions with continuous predictors. It is this functionality that allowed the analysis of the reproductive cycles of broadcast spawning gastropods, which involved interactions between sex, species, and the cyclic smooths, an analysis that would not have been possible using dredge. While we have not been able to provide a relevant case study here, our full‐subsets function may also prove useful in the case of exploring the importance of lagged predictors (Fulton et al., 2014). Lagged variables could all be passed to the function for model construction, with automatic removal of models including more than one, as they would almost certainly be highly correlated.

The function allows exploration of a wide range of potentially correlated predictors simultaneously (models containing correlated predictors are removed from the candidate set, but all variables are still included in the model set for evaluation). This can be useful in situations where there is considerable uncertainty regarding which of several correlated variables might be the best predictors and is particularly relevant where the main aim of a study is exploration. However, inclusion of sets of highly correlated variables can weaken interpretation of results, as this can split the “importance” scores across a range of metrics representing a single ecosystem driver. While it may be possible to sum importance scores across the included metrics to derive an overall importance score for the ecosystem driver of interest, it is better to avoid including variables that effectively measure the same underlying functional process. While included predictor variables can be correlated with one another using our full‐subsets approach (as models containing correlated predictors are automatically removed from the model set), it is up to the ecologist to ensure only variables that have a sensible reason for being considered in the model set are included. Note also that while we have used simple bivariate correlations to identify possible collinearities, which is appropriate given the relatively low complexity models we recommend here (default number of maximum included predictors = 3). If many predictors are to be included within individual models in the candidate set, more complex collinearities may exist. If this is the case, we recommend the user make use of a range of more complex diagnostic tools for evaluating collinearity (Dorman et al. 2012) that are readily implementable via existing R packages (e.g., Hendrikx 2012) to construct their own bivariate inclusion matrix to be passed to the function (see Appendix S1, cor.matrix).

As with any statistical methodology that becomes widely used in ecology, there is considerable scope for misuse of information theoretical approaches, with the most pertinent issues covered by Anderson and Burnham (2002). A concern of computer algorithm approaches for building model sets for comparison within the information theoretic framework is that it leads to analyses based on poor science questions and too many models, without careful consideration of the science issues being captured. This is a valid criticism, and it is certainly true that the full‐subsets function presented here can be easily misused in this way. Our initial motivation for the development of an automated approach for the construction of model sets stemmed from the fact that manually building complete models sets can be both tedious and prone to error (often potentially valid candidate models are missed). The full‐subsets function described here provides a balance between tedious manual coding of all candidate models and convenient automation of the most likely useful candidate model set. We provide considerable flexibility in our function aimed at ensuring only scientifically sensible, and valid models are included in the final set, including (1) the capacity to remove models containing correlated predictors, which often yield spurious results and are scientifically indefensible; (2) restriction of the maximum number of included predictors to ensure that included models remain ecologically interpretable; (3) limitation of *k* in GAM models, such that models with overly complex relationships between predictor and response variables are not considered; (4) the capacity to include “null” terms in all models, where there are clear known relationships that must be included for valid inference; and (5) the ability to restrict factor and smooth interactions to only those that are sensible and scientifically relevant. In addition to these features, we highly recommend that careful consideration be given to the included predictors, both continuous and categorical. These should be restricted to those that have a reasonable scientific basis for being of relevance to the response. In addition, the fitted model set should be screened carefully to ensure that all are sensible and that potentially important models have not been excluded. Users must also be mindful that a full‐subsets approach is not always the best solution to analyzing a large range of predictors. There are clearly times where data reduction techniques (such as PCA) are useful, such as when there is no theoretical reason to understand how (or expect) a single predictor to be an important driver. Another criticism is that full‐subsets approaches applied to observational datasets can only highlight where there are strong and weak relationships and do not imply causality. While the methods are useful where the aim is primarily exploring and elucidating important relationships that can help build hypotheses and theoretical models, experimental studies are generally required to properly establish cause and effect pathways.

Finally, we encourage users of our function to fully embrace the value of information theoretic approaches, rather than using these simply as an alternative model selection tool aimed at yielding a most “parsimonious” or “best” model. There is inherent value in the ability to explore the relative importance of predictors, as we have focused on in our case studies. The function outputs summed Akaike weights as a metric of variable importance, a widely used measure in ecology (Grueber, Nakagawa, Laws, & Jamieson, 2011), but which has come under recent criticism (Galipaud, Gillingham, David, & Dechaume‐Moncharmont, 2014; Galipaud, Gillingham, & Dechaume‐Moncharmont, 2017; but see Giam and Olden 2016). A range of other metrics may also be considered for assigning variable importance, such as model averaged standardized parameter estimates (Galipaud et al., 2017) or methods focused on assessing dispersion importance (Grömping, 2006); however, these are not currently available for GAM model fits and cannot therefore be easily implemented here. In the meantime, we urge caution in the interpretation of summed AIC weights and encourage readers to be aware of common misconceptions regarding their use in ecology (Galipaud et al., 2014). Importantly, aside from outputting variable importance scores, our function also returns a complete set of fitted models that can be further utilized by the user. For example, information theoretic approaches can yield sets of competing models with relatively similar support. In such cases, this model uncertainty can be properly captured using multimodel inference approaches (Burnham and Anderson 2002), yielding more robust prediction outcomes than single model inference. In addition, the fitted model set can be interrogated and/or incorporated into a number of additional procedures, such as using training and testing subsets to assess model predictive performance.

## CONFLICT OF INTEREST

None declared.

## AUTHOR CONTRIBUTIONS

R.F wrote the R code for the full subsets function; T.J.L, S.K.W, S.M.S, and A.C.L collected the data for the case studies. All authors contributed to case study analyses and draft revisions.

## DATA ACCESSIBILITY

All R code and case study data used in this study are available on github https://github.com/beckyfisher/FSSgam
