# Supplementary material for: A simple function for full‐subsets multiple regression in ecology with R
Source: Ecol Evol. 2018 May 20;8(12):6104–13. doi: 10.1002/ece3.4134 (PMC6024142; doi:10.1002/ece3.4134)
Supplement: Supplementary file 1 [file ECE3-8-6104-s001.docx]

# **Appendix 1.** Full subsets function arguments.

| Argument | Default value | Details |
| --- | --- | --- |
| use.dat | None | A data.frame, with columns matching those included in pred.vars.cont and pred.vars.fact, the response variable to be analysed and any other fields required for the analysis (such as random effects, see **test.fit**). Note that any variables in use.dat that are used in model fits must not contain missing values, as this invalidates comparison via AICc/ BIC. If missing values occur among the predictor variables the function will return an error warning indicating that these rows need to be removed or interpolated. |
| test.fit | None | A gam model fitted via a call to gam (mgcv) or uGamm (MuMIn). This can use any of the (preferably continuous) predictors in the call and will be used as a model to update in the fitting of the model set.  The test fit must contain the appropriate random effects and call to family (if not gaussian) and if gamm4 should be used, or gamm in the case of a uGamm call (see ?uGamm).  Both gamm from mgcv and gamm4 have slightly different features, as well as advantages and disadvantages, thus it is important that the full subsets function is able to deal with test.fit models based on either package. For example gamm4 is based on the lme4 package [Bates, D.M. (2010) lme4: Mixed-Effects Modeling with R. Springer, New York] which allows crossed random effects and avoids issues with PQL for non-gaussian model fits. On the other hand gamm (mgcv) is based on nlme which allows correlation structures [Box, G.E.P., Jenkins, G.M., and Reinsel G.C. (1994) "Time Series Analysis: Forecasting and Control", 3rd Edition, Holden-Day], variance structures [Pinheiro, J.C. and Bates., D.M. (1996) "Unconstrained Parametrizations for Variance-Covariance Matrices", Statistics and Computing, 6, 289-296], and a broader range of families that are not yet available in lmer (see ?family.mgcv).  Models that have no random effects and are based only on gam (mgcv) are best fit via a direct call to gam, rather than using the uGamm wrapper. |
| pred.vars.cont | None | A character vector indicating the continuous predictors to use. By default all continuous predictors will be fitted using a smoother (but see argument **linear.vars**). These must match column names in **use.dat** exactly. If NA is used the function can be run without any smooth predictors. |
| pred.vars.fact | NA | NA if there are no factor predictors, or a character vector indicating the factor predictors to use. These must match column names in **use.dat** exactly. All pred.vars.fact variables will be coerced to a factor, with unused levels simultaneously dropped via a call to factor. By default all factor variables will be included as interactions with the continuous smoothers via their specification as a by argument in the call to gam (but see **factor.smooth.interactions**). Factor interactions are not included by default (but see **factor.factor.interactions**). |
| factor.factor.interactions | F | A logical value indicating if interactions between factors should be included, or only their main effects. Defaults to FALSE. Note that this can substantially increase the number of models in the candidate set. Not recommended when there are factors with many levels. Alternatively character vector specifying which factor predictors to include as interactions. These must be contained within pred.vars.fact.  If factor.factor.interactions is set to TRUE the function automatically generate “hard coded” interaction variables up to the maximum number of predictors (see “max.predictors” below) using combn. New factors are generated by pasting the resulting unique combinations together. This method of generating interaction terms is necessary because smooth-factor interactions are specified as “by” arguments in calls to gam(m4). Because the full subsets function automatically checks for collinearity, there is no issue with constructing model sets with multiple factor arguments that are higher order factors of each other, as these are invariably collinear and subsequently removed (see cov.cutoff below). If a user defined cor.matrix is passed to the function (see cor.matrix) this must include these hard coded interactions. |
| smooth.smooth..interactions | F | A logical value indicating if the function should include te smooths of second order continuous predictor interactions. If set to TRUE, all continuous predictors will be combined as bivariate calls to “te”. Alternatively character vector specifying which continuous predictors to include. These must be contained within pred.vars.cont. |
| factor.smooth.interactions | pred.vars.fact | A character vector specifying if/which factor variables should be included as "by" argument interaction terms with the continuous smooth predictors. Default is the list of **pred.vars.fact**, meaning that all will be included by default. If factor.factor.interactions is TRUE, factor interactions variables will also be included as “by” arguments, yielding higher order interactions up to the specified model max.predictors. If a character vector is specified, only those contained in the character vector will be included. Note that specified factors must also be included in **pred.vars.fact**. If specified as NA no factor interactions will be included. |
| max.predictors | 3 | An integer indicating the maximum number of predictors to include in any one model. |
| cov.cutoff | 0.28 | A numeric value between 0 and 1 indicating the correlation cutoff value to use for excluding collinear models, based on the cor.matrix (see below). The default value is 0.28 (see Graham MH (2003). It is highly recommended to keep this value low, as correlation among predictors can yield spurious results. Note that predictors with a correlation greater than the specified value will still appear in the model set but will never appear in the same model. Including highly correlated predictors can make interpreting variable importance values difficult. |
| cor.matrix | NA | By default predictor correlations are evaluated via a call to check.correlations, a function taking a data.frame (containing all predictors) as argument and generating a correlation matrix comprised of: 1) correlation coefficients between all continuous predictors via a call to cor; 2) approximate correlation values between continuous predictors and factors, as the square-route of the R^2^ value obtained via a call to lm, where the continuous predictor is modelled as a response and the factor variable as a single fixed factor; and 3) approximate correlations values between factor predictors, as the square-route of the R^2^ value obtained via a call multinom [(from package nnet, Venables & Ripley 2002)](https://paperpile.com/c/8mZnHu/HdlW/?prefix=from%20package%20nnet%2C). Note that any user constructed pairwise matrix can be passed to the function and used for pairwise exclusion of variables from individual models. |
| k | 5 | An integer indicating the dimension of the basis used to represent the smooth term (see ?s). The default value is 5. Higher values are not recommended unless a complex trend between the response variable and the continuous predictor variables is expected, and the data are sufficient to support this. k can be reduced to as low as 3 where there is trouble obtaining convergence, or sample size is low. Note that this must be set to override the default value, regardless of what k is used in the **test.fit** |
| bs.arg | 'cr' | Specification of the smoother to use, see ?s for more information on smoother provided in gam (mgcv). Note that all continuous predictors specified in **pred.vars.cont** will be fitted using the same smooth, unless they are also specified as **linear.terms** or **cyclic.vars**. Note that any specification of bs in **test.fit** is discarded. |
| cyclic.vars | NA | NA if there are no cyclic predictors, or if there are cyclic predictors,a character vector containing the names of any of the continuous predictors that should be modelled as cyclic variables. Note that these must also be contained in the **pred.vars.cont** charactervector. Please also note there are issues with bs='cc' and model selection as this uses by default shrinkage. With shrinkage, variables are retained in models but with zero edf, which makes interpretation of AICc and BIC confusing. To account for this always select only the most parsimonious model (that with the fewest parameters), not just that with the lowest AICc. Reported estimated degrees of freedom (edf) in the model output table represent the sum of the edf of the smooth terms plus the number of parametric coefficients. When cyclic variables are included and shrinkage is used, any estimated edf of the smooth terms that are less than 1 are reset to 1 before summing to ensure the the total number of predictors in the model is captured properly. |
| linear.vars | NA | NA if there are no continuous predictors to be treated as linear (not fitted as smooths). Only use this where variables are clearly continuous in nature, but you are confident a linear relationship is valid. It may also be useful for continuous predictors that are not well distributed along the x-axis (ie, sampling was conducted in clumped distances from a feature of interest). Where this is necessary, transformations should be considered where they can be used to theoretically "linearize" response relationships. |
| null.terms | “” | A character vector indicating the form of any re smooths to be included in gam [e.g. "s(site,bs='re')"] or any other fixed terms or smooths that the user wants to include in the null model. Use of bs="re" is an alternative way of fitting simple random structures that avoids use of PQL and allows a the greater range of families available in gam.mgcv to be used. see ?s and links therein. Note: make sure you use "gam" instead of uGamm to make sure PQL is not used. |
| parallel | F | A logical value indicating if parallel processing should be used. The default is FALSE. Before changing to TRUE, please make sure parallel processing works for you by running the code: cl=makePSOCKcluster(2); registerDoParallel(cl) |
| n.cores | 4 | The number of cores to use in the parallel processing when **parallel** is set to T. The default value is 4, which is suitable for most current quad core machines. |
| max.models | 500 | The total number of models allowed to be fit. If the candidate set is bigger than this value, an error message will be returned, asking the user to reset this to a larger value. |
| r2.type | "r2.lm.est | The value to extract from the gam model fit to use as the R squared value. Defaults to "r2.lm.est" which returns and estimated R squared value based on a linear regression between the observed and predicted values. "r2" will return the adjusted R.sq as reported by gam, gamm or gamm4."dev" will return the deviance explained as reported by gam or gamm. Note gamm4 does not currently return a deviance. |
| report.unique.r2 | F | The estimated null model R2 is subtracted from each model R2 to give an idea of the unique variance explained. This can be useful where null terms are included in the model set. |
