# Supplementary material for: A simple function for full‐subsets multiple regression in ecology with R
Source: Ecol Evol. 2018 May 20;8(12):6104–13. doi: 10.1002/ece3.4134 (PMC6024142; doi:10.1002/ece3.4134)
Supplement: Supplementary file 2 [file ECE3-8-6104-s002.docx]

# **Appendix 2.** Full subsets function outputs.

| Output | Details | |
| --- | --- | --- |
| $mod.data.out | A data.frame that contains the statistics associated with each model fit. This includes AICc and BIC, delta values (e.g. AICc-(min(AICc)), corresponding weight (ωi) values [(Burnham and Anderson 2003)](https://paperpile.com/c/8mZnHu/whqy), an estimate of the model R^2^, and a column for each of the included predictor variables containing either 0 (variable not included in the model) or 1 (variable is present in the model).  Use of BIC in information theoretic approaches has been heavily criticised because of the inherent assumption of BIC that there is a “true” model that is represented in the candidate set (Anderson & Burnham 2002). Rather than decide a-priori which model selection tool users should adopt, we supply both as part of the function outputs.  To simplify output, only AICc and AICc based model weights, rather than AIC, are included as these are asymptotically equivalent at large sample sizes, and for small sample sizes AICc should be used in any case.  Calculating R^2^ values is non-trivial for mixed models, especially non-gaussian cases (and some argue should not be done at all). We have supplied a range of methods for estimating R^2^ (r2.type, Appendix 1), as in our experience a single method rarely performs adequately across all scenarios. | |
| $used.data | A data.frame which is identical to the data.frame initially supplied by the user, but with any hard coded interaction terms appended via cbind. | |
| $predictor.correlations | The matrix of estimated predictor correlations returned by the function check.correlations and used for model exclusion based on cov.cutoff (supplementary Appendix 1). | |
| $failed.models | A list containing the try-error catch associated with models that failed to fit. Ideally the list of failed models should be empty, but when this is not the case interrogating failed.models provides a useful means of troubleshooting. Users can examine which models are not fitting and explore the reasons for this by fitting the failed models outside the full.subsets.gam call based on the listed formula. When a large number of models fail to fit properly it usually indicates poor specification of the initial test.fit (supplementary Appendix 1) or other arguments in the call to full.subsets.gam (such as the inclusion of factor interactions when there are few data within each level of the factor), or that inappropriate variables are being included in the model set. | |
| $success.models | A complete list of all successfully fitted models. This can be used for multimodel inference and creating model averaged predictions. | |
| $variable.importance | A list containing importance scores for each included predictor.  To determine the relative importance of each predictor across the whole model set we summed the ωi values for all models containing each variable. The higher the combined weights for an explanatory parameter, the more important it is in the analysis (Burnham & Anderson, 2002). An assumption of the use of summed model weights to infer variable importance is that the number of models in which the different predictors are present is uniform. As our function removes models with correlated predictors, this is not always the case. To overcome this issue, the summed variable.importance scores are the summed weights for the best n models, where n is equal to the minimum number of models any one predictor is present in. | |
|  | $aic | Variable importance values based on summed AICc weights |
|  | $bic | Variable importance values based on summed BIC weights. |
