# Supplementary material for: A simple function for full‐subsets multiple regression in ecology with R
Source: Ecol Evol. 2018 May 20;8(12):6104–13. doi: 10.1002/ece3.4134 (PMC6024142; doi:10.1002/ece3.4134)
Supplement: Supplementary file 3 [file ECE3-8-6104-s003.docx]

# Case Study 1: The relative influence of management and habitat on fish abundance and biomass

## Background

Coral reef fish are highly diverse assemblages that provide important ecosystem services for millions of people [(Pratchett *et al.* 2014)](https://paperpile.com/c/7DszSz/1kMC). These services are however threatened by overfishing [(Newton *et al.* 2007; MacNeil *et al.* 2015)](https://paperpile.com/c/7DszSz/YTLT+3s2A) and a loss of habitat, in particular corals [(Wilson *et al.* 2006)](https://paperpile.com/c/7DszSz/rxEK) and the structure they provide [(Rogers *et al.* 2014)](https://paperpile.com/c/7DszSz/DS9U). No-take reserves (NTR) promote higher abundance and biomass of fish [(Russ 2002; McClanahan *et al.* 2009)](https://paperpile.com/c/7DszSz/gTrT+2PR5) and conserve ecosystem function [(Graham *et al.* 2011)](https://paperpile.com/c/7DszSz/1YyA). It is clear that NTR cannot prevent large scale disturbances, such as heat stress that cause extensive coral loss and decline in fish [(Jones *et al.* 2004; Graham *et al.* 2008)](https://paperpile.com/c/7DszSz/WiIz+dGXP), however a reduction in local pressures in NTR may facilitate greater resilience of coral reefs [(Hughes *et al.* 2010)](https://paperpile.com/c/7DszSz/KwFP).

By examining patch reefs of differing habitat quality inside and outside of NTR within the Ningaloo marine park, Wilson et al. [(2012)](https://paperpile.com/c/7DszSz/lIuD/?noauthor=1) explored how habitat degradation and fishing influenced the abundance and biomass of fish from different functional groups. Explanatory variables in the original analyses were summarised using the scores from the two axes of a principal components analysis (PCA), making it was impossible to tease apart the relative importance of variables that were correlated along the axis. The revised analyses here based on a full subsets multiple regression approach allows the influence of habitat variables to be assessed independently.

## Methods

Benthic and fish communities on patch reefs within fished (n=35) and NTR (n=33) of the Ningaloo lagoon were surveyed to investigate the influence of habitat and fishing on coral reef fish. On each patch the seascape level of reef structure (complexity) was assessed using a six point scale, where 0 represents no vertical relief and 5 represents highly complex reefs with small overhangs and caves [(Polunin & Roberts 1993)](https://paperpile.com/c/7DszSz/0dkp). Finer scale physical reef structure (rugosity) was measured as the linear distance covered by a 3m length of chain fitted to the reef contour [(Risk 1972)](https://paperpile.com/c/7DszSz/oSKS). Benthic cover was quantified along a 5m line intercept transect using the categories: high complex corals such as Branching morphologies (HC), low complexity corals such as massive and encrusting morphologies (LC), and macroalgae (Macro).

Abundance and size of adult fish was estimated by a diver using a point count method. Large mobile fish were initially counted from the perimeter of the patch, after which the entire patch was surveyed for smaller site attached species. Fish were placed into broad functional groups based on their diets and biomass calculated from length weight relationships [(Froese & Pauly 2011)](https://paperpile.com/c/7DszSz/iGKm).

### Data analysis

A generalised additive model with full subsets analyses was used to determine if fishing, habitat or interactions between these variables best explained variance in abundance and biomass of functional fish groups. In the original analysis PCA was used to summarise habitat information and scores from principal components 1 and 2 were included in the full subsets analysis. Principal component 1 was positively correlated with complexity and rugosity, but negatively correlated with percent macroalgal cover, while principal component 2 was positively correlated with HC, but negatively correlated with LC. In the revised analysis scores from both principal components (Score1 and Score2) were again included as explanatory variables, along with percent cover estimates from line intercept transects (HC, LC, Macro), complexity and rugosity measures. Zone (fished or NTR) was included as an assessment of fishing pressure. The motivation for including the original PCA scores in the revised analysis was that this should provide a means of comparing if the raw habitat predictors have a stronger relationship with the fish abundance and biomass variables than the PCA aggregate. Both abundance and biomass data were modelled using a tweedie distribution, implemented via a call to gam (mgcv) with the random effect of site included using the bs=’re’ specification. Smoothers to both square-route surface area of the patch reef as well as depth were included in all models (and as part of the null model) via the null.terms argument of the full subsets gam function. Given the complexity of the null model, the maximum number of additional predictors were limited to only an additional two terms (max.predictors=2) and k was limited to 3 to enforce strictly monotonic relationships. All R code and the dataset used in analysis can be found at:

<https://github.com/beckyfisher/FSSgam/blob/master/case_study1_reef_fish.R>.

## Results and discussion

Seascape measures of patch reef complexity were generally the best predictor of both fish abundance and biomass (Table A3.1, Figure A3.1). Fish abundance and biomass were low on reefs with no relief and were higher on structurally complex reefs (Fig A3.2). These findings are consistent with other studies that show measures of seascape complexity are positively correlated with fish abundance, and often outperform other measures of complexity [(Wilson *et al.* 2007; Collins *et al.* 2016)](https://paperpile.com/c/7DszSz/WbOe+zciz). The results generally support the original findings of Wilson et al.[(2012)](https://paperpile.com/c/7DszSz/lIuD/?noauthor=1) that there are strong relationships with habitat and only weak evidence for an effect of the zoning status. However, here we were able to tease apart the influence of correlated habitat variables. For example, the revised analysis found herbivore abundance was positively correlated with complexity, which is similar to recent studies at Ningaloo that also found abundance of these fish is not closely related to macroalgal cover [(Wilson *et al.* 2014)](https://paperpile.com/c/7DszSz/eRos) and is better explained by reef structure [(Downie *et al.* 2013)](https://paperpile.com/c/7DszSz/hCf0). Conversely, Wilson et al. [(2012)](https://paperpile.com/c/7DszSz/lIuD/?noauthor=1) identified scores from principal component 1 as the best predictor of herbivore abundances which inferred a positive effect of both rugosity and complexity and negative effect of macroalgal cover.

**Table A3.1.** Summary results from complete subsets analyses showing all models within 2 AICc of the best model, estimated degrees of freedom (edf), ∆AICc, AICc weights and R^2^ estimates.

| Response | Model | AICc | BIC | ΔAICc | ΔBIC | wi.AICc | wi.BIC | R2 | edf |
| --- | --- | --- | --- | --- | --- | --- | --- | --- | --- |
| Herbivore abundance | complexity | 517.91 | 532.44 | 0 | 0 | 0.53 | 0.77 | 0.6 | 6.22 |
|  | complexity+SCORE2 | 519.87 | 536.42 | 1.96 | 3.98 | 0.2 | 0.11 | 0.6 | 7.37 |
| Invertivore abundance | complexity | 609.45 | 630.52 | 0 | 0 | 0.31 | 0.38 | 0.53 | 8.58 |
|  | complexity+ZONE | 609.51 | 630.84 | 0.06 | 0.32 | 0.3 | 0.32 | 0.53 | 9.04 |
|  | complexity+SCORE2 | 609.55 | 631.58 | 0.11 | 1.07 | 0.29 | 0.22 | 0.53 | 9.35 |
| Piscivore abundance | complexity+ZONE | 459.01 | 475.33 | 0 | 0 | 0.24 | 0.32 | 0.32 | 6.01 |
|  | complexity | 459.09 | 475.36 | 0.08 | 0.03 | 0.23 | 0.31 | 0.32 | 5.64 |
|  | ZONE+SCORE1.by.ZONE | 459.98 | 478.4 | 0.97 | 3.07 | 0.15 | 0.07 | 0.3 | 7.86 |
|  | ZONE+complexity.by.ZONE | 460.17 | 477.04 | 1.16 | 1.71 | 0.13 | 0.13 | 0.3 | 7 |
| Planktivore abundance | SCORE1 | 687.56 | 702.78 | 0 | 0 | 0.43 | 0.65 | 0.33 | 6.72 |
| Herbivore biomass | complexity | 1194.11 | 1208.69 | 0 | 0 | 0.64 | 0.81 | 0.37 | 6.2 |
| Invertivore biomass | complexity | 1236.49 | 1251.38 | 0 | 0 | 0.47 | 0.71 | 0.41 | 6.42 |
|  | complexity+SCORE2 | 1238.44 | 1254.86 | 1.95 | 3.48 | 0.18 | 0.12 | 0.41 | 7.41 |
| Piscivore biomass | SCORE1 | 1025.67 | 1037.54 | 0 | 0 | 0.28 | 0.43 | 0.29 | 5.05 |
|  | complexity | 1026.17 | 1037.89 | 0.5 | 0.34 | 0.22 | 0.36 | 0.3 | 5 |
| Planktivore biomass | complexity+ZONE | 1012.27 | 1033.93 | 0 | 0 | 0.47 | 0.49 | 0.33 | 9.65 |
|  | complexity | 1012.75 | 1034.27 | 0.48 | 0.34 | 0.37 | 0.41 | 0.32 | 9.31 |

| 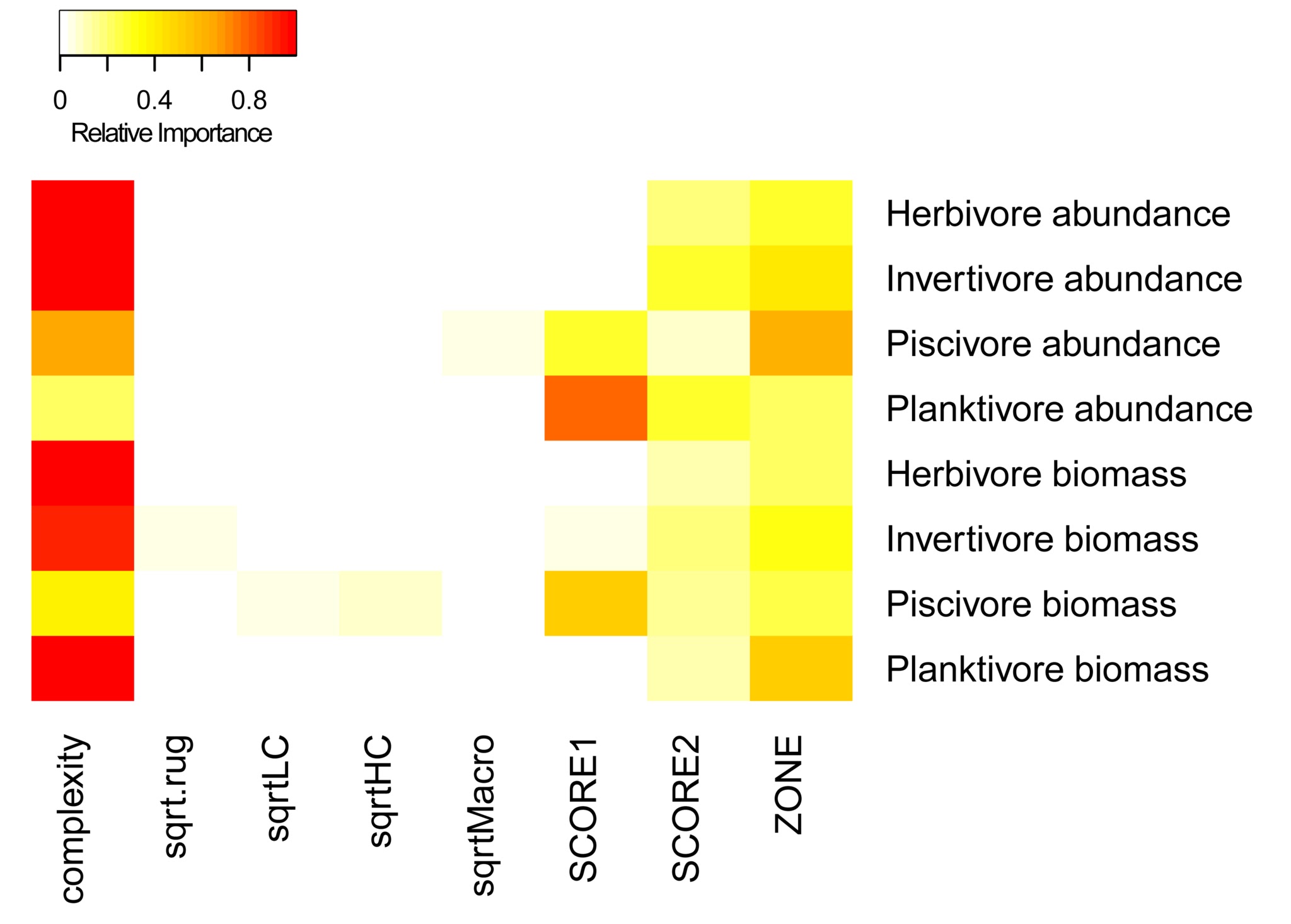 | **Fig. A3.1.** Variable importance scores from full subsets GAMM analyses exploring the relationship between habitat variables and abundance and biomass four functional fish feeding guilds (see Table A3.1). |
| --- | --- |

Interestingly, the abundance of planktivores and piscivores was positively related to scores from principal component 1 (Figs A3.1& A3.2), suggesting summarised metrics of habitat from PCA may still be relevant to some components of the fish assemblage. A habitat descriptor that incorporates multiple correlated factors may be more important if there is a diverse array of habitat associations among fish within a trophic guild. For example, the planktivores include small bodied damselfish that have close associations with complex corals [(Coker *et al.* 2014)](https://paperpile.com/c/7DszSz/osHQ), cardinalfish that are often found in caves or branching corals [(Marnane & Bellwood 2002)](https://paperpile.com/c/7DszSz/ypWp), and larger fish that are likely transient and more associated with seascape measures of habitat [(Friedlander & Parrish 1998)](https://paperpile.com/c/7DszSz/uj9S). As large bodied fish also typically contribute more to community biomass than their small counterparts [(Ackerman & Bellwood 2000)](https://paperpile.com/c/7DszSz/zWVO) it is not surprising that planktivore biomass is more closely aligned with seascape complexity than other measures of habitat.

Overall the results of the revised analysis are similar to those from the original study based on the use of data-reduction via PCA. However, by including the underlying habitat information, the new analysis provides a clearer picture of which elements of the benthic assemblage are most important to fish. This additional information is useful for building scientific hypotheses and parametric functional models for how such fishes may be influenced by changes in habitat [(such as is expected due to ocean warming and climate change, see Pratchett *et al.* 2015)](https://paperpile.com/c/7DszSz/NvVt/?prefix=such%20as%20is%20expected%20due%20to%20ocean%20warming%20and%20climate%20change%2C%20see), as well as informing which elements of the habitat should be a focus for management targets.


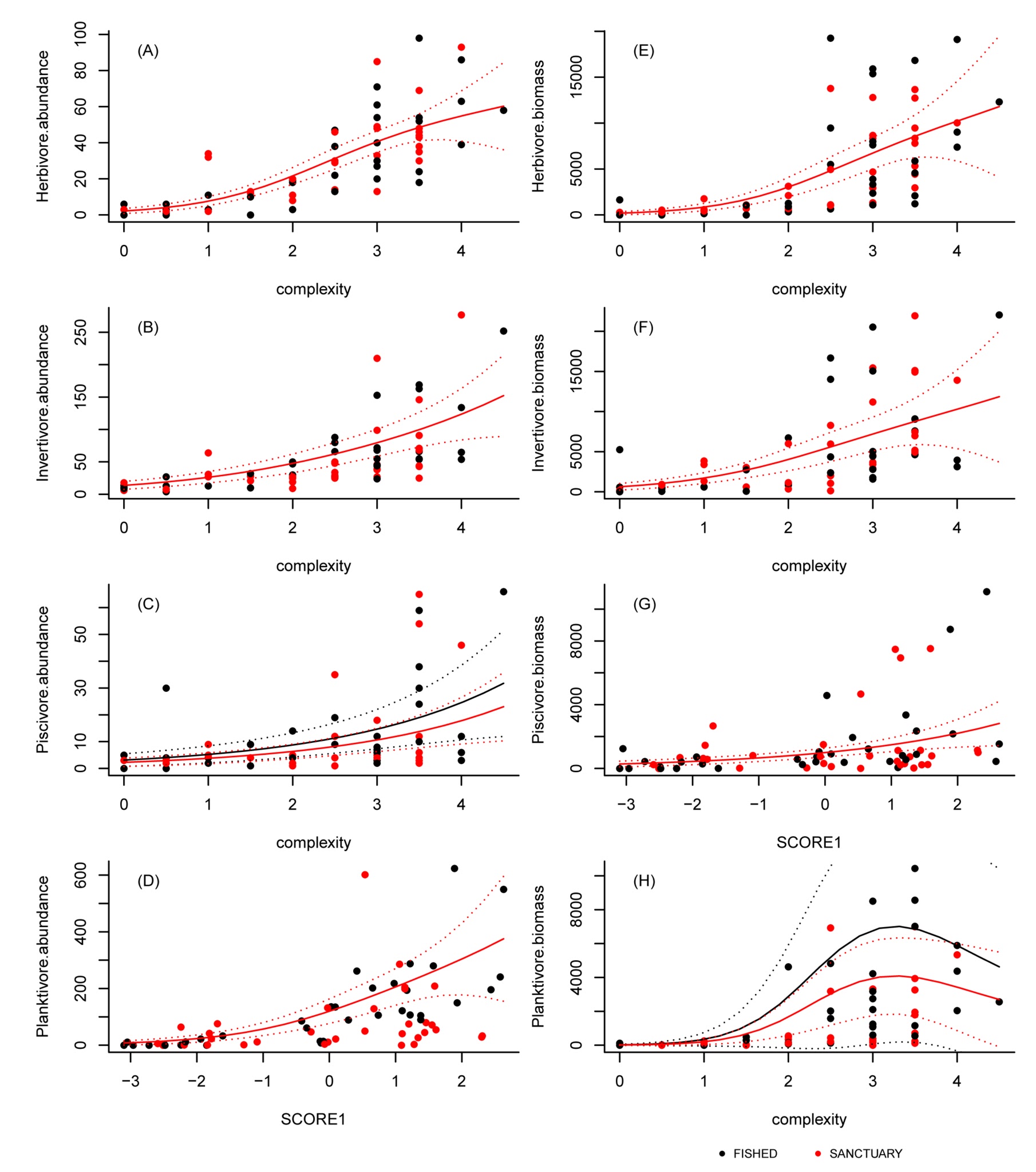


**Fig. A3.2.** Abundance (A-D) and biomass (E-H) of four functional fish feeding guilds and their relationships with the habitat predictor present in the fitted model with the lowest AICc value (see Table A3.1). Coloured symbols indicate data from the fished and sanctuary zones. Solid lines are fitted gam curves, with dashed lines indicate 95% confidence bands.

## References

[Ackerman, J.L. & Bellwood, D.R. (2000). Reef fish assemblages: a re-evaluation using enclosed rotenone stations. *Marine Ecology Progress Series*, **206**, 11.](http://paperpile.com/b/7DszSz/zWVO)

[Coker, D.J., Wilson, S.K. & Pratchett, M.S. (2014). Importance of live coral habitat for reef fishes. *Reviews in Fish Biology and Fisheries*, **24**, 89–126.](http://paperpile.com/b/7DszSz/osHQ)

[Collins, D.L., Langlois, T.J., Bond, T., Holmes, T.H., Harvey, E.S., Fisher, R. & McLean, D.L. (2016). A novel stereo-video method to investigate fish-habitat relationships. *Methods in Ecology and Evolution*, **8**, 116–125.](http://paperpile.com/b/7DszSz/zciz)

[Downie, R.A., Babcock, R.C., Thomson, D.P. & Vanderklift, M.A. (2013). Density of herbivorous fish and intensity of herbivory are influenced by proximity to coral reefs. *Marine Ecology Progress Series*, **482**, 217–225.](http://paperpile.com/b/7DszSz/hCf0)

[Friedlander, A.M. & Parrish, J.D. (1998). Habitat characteristics affecting fish assemblages on a Hawaiian coral reef. *Journal of Experimental Marine Biology and Ecology*, **224**, 1–30.](http://paperpile.com/b/7DszSz/uj9S)

[Froese, R. & Pauly, D. (2011). *FishBase*. www.fishbase.org, last accessed 31/03/2011.](http://paperpile.com/b/7DszSz/iGKm)

[Graham, N.A.J., Ainsworth, T.D., Baird, A.H., Ban, N.C., Bay, L.K., Cinner, J.E., De Freitas, D.M., Diaz-Pulido, G., Dornelas, M., Dunn, S.R. & Others. (2011). From microbes to people: tractable benefits of no-take areas for coral reefs. *Oceanography and Marine Biology-an Annual Review*, **49**, 105.](http://paperpile.com/b/7DszSz/1YyA)

[Graham, N.A.J., McClanahan, T.R., MacNeil, M.A., Wilson, S.K., Polunin, N.V.C., Jennings, S., Chabanet, P., Clark, S., Spalding, M.D., Letourneur, Y., Bigot, L., Galzin, R., Ohman, M.C., Garpe, K.C., Edwards, A.J. & Sheppard, C.R.C. (2008). Climate warming, marine protected areas and the ocean-scale integrity of coral reef ecosystems. *PloS One*, **3**, e3039.](http://paperpile.com/b/7DszSz/dGXP)

[Hughes, T.P., Graham, N.A.J., Jackson, J.B.C., Mumby, P.J. & Steneck, R.S. (2010). Rising to the challenge of sustaining coral reef resilience. *Trends in Ecology & Evolution*, **25**, 633–642.](http://paperpile.com/b/7DszSz/KwFP)

[Jones, G.P., McCormick, M.I., Srinivasan, M. & Eagle, J.V. (2004). Coral decline threatens fish biodiversity in marine reserves. *Proceedings of the National Academy of Sciences of the United States of America*, **101**, 8251–8253.](http://paperpile.com/b/7DszSz/WiIz)

[MacNeil, M.A., Graham, N.A.J., Cinner, J.E., Wilson, S.K., Williams, I.D., Maina, J., Newman, S., Friedlander, A.M., Jupiter, S., Polunin, N.V.C. & McClanahan, T.R. (2015). Recovery potential of the world’s coral reef fishes. *Nature*, **520**, 341–344.](http://paperpile.com/b/7DszSz/3s2A)

[Marnane, M.J. & Bellwood, D.R. (2002). Diet and nocturnal foraging in cardinalfishes (Apogonidae) at One Tree Reef, Great Barrier Reef, Australia. *Marine Ecology Progress Series*, **231**, 261–268.](http://paperpile.com/b/7DszSz/ypWp)

[McClanahan, T.R., Graham, N.A.J., Wilson, S.K., Letourneur, Y. & Fisher, R. (2009). Effects of fisheries closure size, age, and history of compliance on coral reef fish communities in the western Indian Ocean. *Marine Ecology Progress Series*, **396**, 99–109.](http://paperpile.com/b/7DszSz/2PR5)

[Newton, K., Côté, I.M., Pilling, G.M., Jennings, S. & Dulvy, N.K. (2007). Current and future sustainability of island coral reef fisheries. *Current Biology*, **17**, 655–658.](http://paperpile.com/b/7DszSz/YTLT)

[Polunin, N. & Roberts, C.M. (1993). Greater biomass and value of target coral-reef fishes in two small Caribbean marine reserves. *Marine Ecology Progress Series*, **100**, 167–176.](http://paperpile.com/b/7DszSz/0dkp)

[Pratchett, M.S., Hoey, A.S. & Wilson, S.K. (2014). Reef degradation and the loss of critical ecosystem goods and services provided by coral reef fishes. *Current Opinion in Environmental Sustainability*, **7**, 37–43.](http://paperpile.com/b/7DszSz/1kMC)

[Pratchett, M.S., Wilson, S.K. & Munday, P.L. (2015). Effects of climate change on coral reef fishes. *Ecology of Fishes on Coral Reefs*, pp. 127–135. Cambridge University Press, Cambridge University Press University Printing House Shaftesbury Road Cambridge CB2 8BS United Kingdom.](http://paperpile.com/b/7DszSz/NvVt)

[Risk, M.J. (1972). Fish diversity on a coral reef in the Virgin Islands. *Atoll Research Bulletin*, **153**, 1–6.](http://paperpile.com/b/7DszSz/oSKS)

[Rogers, A., Blanchard, J.L. & Mumby, P.J. (2014). Vulnerability of coral reef fisheries to a loss of structural complexity. *Current Biology*, **24**, 1000–1005.](http://paperpile.com/b/7DszSz/DS9U)

[Russ, G.R. (2002). Yet another review of Marine Reserves as Reef Fishery Management Tools. *Coral Reef Fishes*, pp. 421–443. Elsevier Science.](http://paperpile.com/b/7DszSz/gTrT)

[Wilson, S.K., Babcock, R.C., Fisher, R., Holmes, T.H., Moore, J.A.Y. & Thomson, D.P. (2012). Relative and combined effects of habitat and fishing on reef fish communities across a limited fishing gradient at Ningaloo. *Marine Environmental Research*, **81**, 1–11.](http://paperpile.com/b/7DszSz/lIuD)

[Wilson, S.K., Fulton, C.J., Depczynski, M., Holmes, T.H., Noble, M.M., Radford, B. & Tinkler, P. (2014). Seasonal changes in habitat structure underpin shifts in macroalgae-associated tropical fish communities. *Marine Biology*, **161**, 2597–2607.](http://paperpile.com/b/7DszSz/eRos)

[Wilson, S.K., Graham, N.A.J. & Polunin, N.V.C. (2007). Appraisal of visual assessments of habitat complexity and benthic composition on coral reefs. *Marine Biology*, **151**, 1069–1076.](http://paperpile.com/b/7DszSz/WbOe)

[Wilson, S.K., Graham, N. & Pratchett, M.S. (2006). Multiple disturbances and the global degradation of coral reefs: are reef fishes at risk or resilient? *Global Change Biology*, **12**, 2220–2234.](http://paperpile.com/b/7DszSz/rxEK)
