# Supplementary material for: A simple function for full‐subsets multiple regression in ecology with R
Source: Ecol Evol. 2018 May 20;8(12):6104–13. doi: 10.1002/ece3.4134 (PMC6024142; doi:10.1002/ece3.4134)
Supplement: Supplementary file 4 [file ECE3-8-6104-s004.docx]

# Case Study 2: The role of large reef-associated predators in structuring adjacent soft-sediment communities

## Background

In ecology, ‘haloes’ have been described in many contexts [(Suchanek 1978; Fairweather 1988)](https://paperpile.com/c/8sOC5X/RLkE+w8bM) and generally are thought to result from a predator or herbivore foraging given distances from a ‘shelter habitat’ out into a ‘food habitat’. Studies in temperate marine ecosystems have found ‘infaunal haloes’, areas of decreased density of soft-sediment fauna adjacent to reefs, at a variety of scales [(0-30 m, Davis *et al.* 1982; 0-70 m, Posey & Ambrose 1994)](https://paperpile.com/c/8sOC5X/AxVk+R46L/?prefix=0-30%20m%2C%20,0-70%20m%2C%20). The model suggesting reef-associated predators are responsible for haloes is supported by studies of gut contents of fish on temperate reefs [(Lindquist *et al.* 1994)](https://paperpile.com/c/8sOC5X/dyDZ). However, convincing demonstrations of predators causing haloes of prey in these systems have been hampered due to limited replication of caging studies [(Posey & Ambrose 1994)](https://paperpile.com/c/8sOC5X/R46L) and the lack of large-scale manipulations [(Thrush *et al.* 2000)](https://paperpile.com/c/8sOC5X/pJGn).

The most conspicuous predators associated with reefs in northeastern New Zealand are the sparid fish *Pagrus auratus* (snapper) and the rock lobster *Jasus edwardsii*. They are highly targeted by local fisheries and occur at higher densities inside no-take marine reserves [(NTR, Babcock *et al.* 1999)](https://paperpile.com/c/8sOC5X/rMkD/?prefix=NTR%2C%20). In this region the influence of predators on rocky reefs has been examined using the existing large-scale experimental framework provided by marine reserves [(Babcock *et al.* 1999; Shears & Babcock 2002)](https://paperpile.com/c/8sOC5X/rMkD+Ehv5). Using this approach, Langlois et al. [(2005)](https://paperpile.com/c/8sOC5X/cQ8I/?noauthor=1) examined the potential role of large reef-associated predators in structuring adjacent soft-sediment communities, by contrasting the densities of predators and prey found inside versus outside three NTR.

Unlike a Before-After-Control-Impact (Underwood 1993) designed study, a potential problem with studies of established NTR is that evidence of a negative relationship between predator densities and densities of prey does not eliminate other potential models [(Hurlbert 1984; Underwood *et al.* 2000)](https://paperpile.com/c/8sOC5X/J4MA+icAl). Results might be confounded by other factors that may structure the soft-sediment community (e.g. wave action, sediment grain-size distributions, organic matter, infaunal interactions). In the original study, Langlois et al. [(2005)](https://paperpile.com/c/8sOC5X/cQ8I/?noauthor=1) hypothesised that predation by large reef-associated predators would result in lower densities of large (> 4 mm) soft-sediment macrofauna inside reserves compared to outside reserves (Predator model). A further hypothesis was that predation would decrease with increasing distances from the reef, resulting in a ‘halo' pattern in the community [(Posey & Ambrose 1994)](https://paperpile.com/c/8sOC5X/R46L), i.e., an increase in prey densities with increasing distances from the reef edge (Distance model).

In the original study, Langlois et al. [(2005)](https://paperpile.com/c/8sOC5X/cQ8I/?noauthor=1) investigated the influence of measured environmental variables (Table A4.1) on the abundance composition of the assemblage inside and outside multiple NTR was investigated using multivariate multiple regression, which found no evidence that any of the measured environmental variables were confounding the comparison of the soft-sediment assemblages inside and outside the multiple NTR. To estimate effects on individual taxa inside and outside the NTR across the multiple random sites and multiple NTR locations, the original analysis used a mixed-model ANOVA, where P-values were obtained using permutations [(Anderson & ter Braak 2003)](https://paperpile.com/c/8sOC5X/u2tj), to account for the great many zeros contained in the data. Any influence of the multiple environmental variables measured (Table A4.1) could not be accounted for in the original analysis of individual taxa due to the limited error families available in GLMM routines at that time. As a consequence it was impossible to tease apart the relative importance of NTR status and distance from the reef edge from variation in snapper or rock lobster density or the influence of measured environmental variables on the individual infaunal taxa. The revised analyses here based on a full subsets multiple regression approach allows the influence of environmental and predator density variables to be assessed independently, whilst accounting for the nesting of random sites inside and outside multiple random NTR and samples collected along a transect of fixed distance from the reef edge.

| 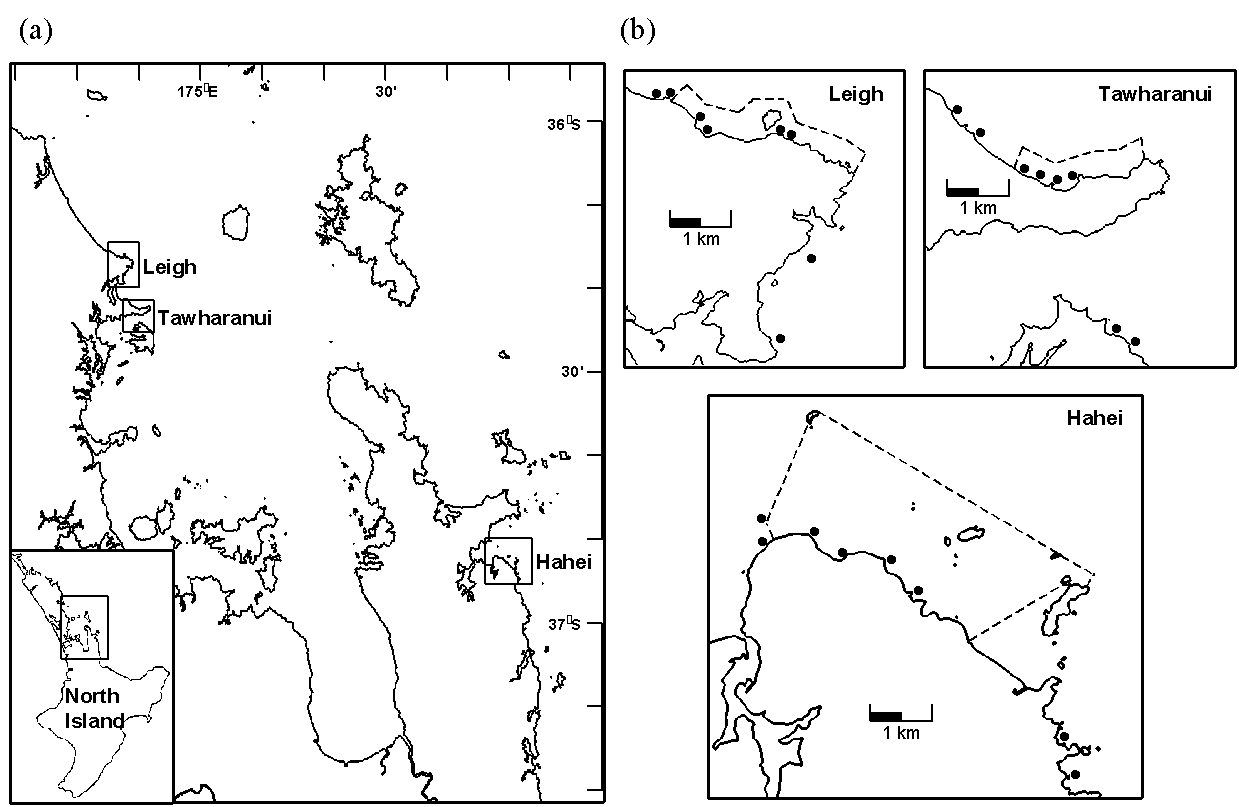 |
| --- |
| **Fig. A4.1.** (a) Map of Hauraki Gulf and environs showing the location of the three reserves surveyed in this study. (b) The insets show the reserve boundaries (dashed lines) and survey sites (solid circles) at the three locations. |

## Methods

In 2002 New Zealand’s northeastern bioregion contained eight reserves, three of which were considered broadly comparable biotype replicates [(Shears & Babcock 2003; Willis *et al.* 2003)](https://paperpile.com/c/8sOC5X/5ZVp+3mkh). This study was carried out between January and March of 2002, using these three locations as a random factor to explicitly test the generality of any potential differences in the effects of marine reserve status [(as in Beck 1997)](https://paperpile.com/c/8sOC5X/GeZr/?prefix=as%20in%20). The Cape Rodney to Okakari Point (Leigh) Marine Reserve (36° 16´S, 174° 48´E) was gazetted in 1975, the Tawharanui Marine Park (36° 22´S, 174° 50´E) was declared a no-take area in 1981 and the Te Whanganui a Hei (Hahei) Marine Reserve (36° 50´S, 175° 49´E) was gazetted in 1993 (Fig. A4.1). At each location eight sites of similar wave exposure and reef / soft-sediment interfaces were chosen, four inside and four outside each marine reserve. To ensure interspersion, in each case, two of the non-reserve sites were located north and the other two located south of the reserve (see Fig. A4.1). The use of three locations and the interspersion of sites on either side of each reserve area was also to mitigate the potential confounding influence of other environmental variables (such as recruitment or food supply). Within each site, sampling was done at each of four distances from the reef edge: 2, 5, 15 and 30 m. These distance strata were comparable to previous studies of infaunal haloes [(Posey & Ambrose 1994)](https://paperpile.com/c/8sOC5X/R46L) and within the likely foraging ranges of snapper [(Parsons *et al.* 2003)](https://paperpile.com/c/8sOC5X/aI5v) and rock lobster [(Kelly *et al.* 1999)](https://paperpile.com/c/8sOC5X/yNlG). At each distance, six replicate samples were obtained using box quadrats measuring 0.5 m2 (1 m x 0.5 m) x 13 cm deep (0.065 m3). Sediment was excavated by hand with a metal scoop and sieved in the field using a sieve with a 4 mm mesh. Other studies from different systems [(Ambrose 1991; Lindquist *et al.* 1994; Posey & Ambrose 1994; Dahlgren *et al.* 1999)](https://paperpile.com/c/8sOC5X/mRoe+dyDZ+R46L+5tXL) have focused on smaller infauna (> 0.5 mm) where the corresponding reef-associated predators were also smaller. In the present study, we focused on larger infauna (> 4 mm), corresponding to the larger reef-associated predators found within reserves [(snapper are ~316 mm mean total length and rock lobster are ~109.9 mm mean carapace length inside reserves, Babcock *et al.* 1999)](https://paperpile.com/c/8sOC5X/rMkD/?prefix=snapper%20are%20~316%20mm%20mean%20total%20length%20and%20rock%20lobster%20are%20~109.9%20mm%20mean%20carapace%20length%20inside%20reserves%2C). Pilot studies showed that large (> 4 mm) fauna in the soft sediment around the chosen locations were relatively patchy in their spatial distribution, thus requiring a sampling unit area of 0.5 m^2^. Organisms retained on the sieve were preserved in 5% formalin and later transferred to 70% ethanol. All organisms were identified to the lowest taxonomic resolution possible. Dry weight abundance estimates were made for all infauna, with the shells of bivalves being excluded from such measurements.

Physical environmental variables measured at each distance stratum at each site included replicate measurements of grain size (n = 3) by dry sieving (with a focus on the proportion of coarse sediments), organic content (n = 3) by ignition at 550°C, bed form measurements (n = 4). Wave exposure was estimated using an index of potential fetch [(Thomas 1986)](https://paperpile.com/c/8sOC5X/fdId). The index was calculated by summing the potential fetch for each 10 degree sector of the compass rose. For open sectors of water the radial distance was arbitrarily set to be 300 km. Estimates of the density of snapper at the reef edge were obtained using baited underwater video (BUV) (n = 4) during April and May 2002 as part of an ongoing monitoring program [(Willis Trevor & Babcock Russell 2000)](https://paperpile.com/c/8sOC5X/lUAk). Estimates of the density of lobster at the reef edge were obtained by underwater visual census (UVC) of 25 m^2^ quadrats (n = 10) during February and March 2002, and the sizes of lobster were estimated using a visual method as described by MacDiarmid [(1991)](https://paperpile.com/c/8sOC5X/C0C3/?noauthor=1). The time discrepancy between the estimates of snapper and rock lobster are not likely to confound any comparison given the relative stability in the temporal variation of these species over summer [(Kelly 2001; Willis *et al.* 2003)](https://paperpile.com/c/8sOC5X/rdw1+3mkh). Densities of octopus and other species known to be predators of soft-sediment fauna were also looked for during rock lobster census dives. From the samples of the infaunal assemblages, the densities of infaunal bioturbators and infaunal predators were also recorded. A list and brief description of the environmental variables measured and subsequently included in analyses are given in Table A4.1.

### Analysis

A generalised additive mixed model with full subsets analyses was used to determine if NTR status, distance from the reef edge, variation in snapper or rock lobster density or the influence of any measured environmental variables or interactions between these predictors best explained variance in abundance of the three most abundant and ubiquitous infaunal taxa. Abundance data were modelled using a tweedie distribution, implemented via a call to gam [(mgcv, Wood 2017)](https://paperpile.com/c/8sOC5X/8Bn0/?prefix=mgcv%2C) with the random effect of site included using the bs=’re’ specification. Smoothers to the random factors of NTR location and nested sites (bs=’re’) were included in all models (and as part of the null model) via the null.terms argument of the full subsets gam function. Given the complexity of the null model, the total number of predictors was limited to only an additional three terms (max.predictors=3) and k was limited to 3. As the density of legal-sized lobster and snapper and NTR status were all relevant to the primary hypothesis, the most parsimonious models within 2 AIC units of the top model that contained any of these variables in addition to any habitat variables were considered as the most parsimonious. The R language for statistical computing [(R Core Team 2017)](https://paperpile.com/c/8sOC5X/sIOd4) was used for all data manipulation [(dplyr, Wickham & Francois 2016; tidyr, Wickham 2017)](https://paperpile.com/c/8sOC5X/zE4a+H4ZZ/?prefix=tidyr%2C,dplyr%2C), analysis [(mgcv, Wood 2011)](https://paperpile.com/c/8sOC5X/7HYk/?prefix=mgcv%2C) and graphing [(ggplot2, Wickham 2009; gridExtra, Auguie 2016)](https://paperpile.com/c/8sOC5X/4T9E+Rd8f/?prefix=ggplot2%2C,gridExtra%2C). All R code and the dataset used in analysis can be found at:<https://github.com/beckyfisher/FSSgam/blob/master/case_study2_soft_sediment.R>

**Table A4.1.** List of environmental variables used as predictors in the full subsets analyses.

| Variable | Description |
| --- | --- |
| Snapper | Density of legal-sized snapper (*Pagrus auratus*) estimated by BUV |
| Rock Lobster | Density of legal-sized rock lobster (*Jasus edwardsii*) estimated by UVC |
| Predatory Infauna | Infauna considered to be predators (see Langlois et al 2005) |
| Bioturbating Infauna | Infauna considered to be bioturbators (see Langlois et al 2005) |
| Grain Size (GS) | Seven variables expressing percentage of grain sizes of ambient sediments (by weight) falling into particular grain-size classes: |
| 125 um | < 0.125 mm |
| 250 um | 0.125 – 0.25 mm |
| 500 um | 0.25 – 0.5 mm |
| 1 mm | 0.5 mm – 1 mm |
| 2 mm | 1 mm – 2mm |
| 4 mm | 2 mm – 4 mm |
| Organics | Sediment organic matter (%) |
| Bed Stress | Estimated using bed form ripple measurements and depth |
| Exposure | Estimation of fetch (km) |
| Depth | Water depth (m) |

## Results and discussion

The most parsimonious model for the bivalve *Dosinia subrosea* included the interaction of distance from reef with NTR status and the 500 um sediment grain size fraction, which (along with random site effects) explained 52% of its distribution (Table A4.2). While fetch occurred in the second top model, importance scores indicated that it was relatively unimportant compared to distance and NTR status (Fig. A4.2). The abundance of *D. subrosea* was negatively correlated with increasing proportion of the 500 um sediment grain size fraction and positively correlated with increasing distance from the reef edge (Fig. A4.3). Subsequent manipulative studies have found that *D. subrosea* are readily preyed upon by the large-bodied rock lobster in the field [(Langlois *et al.* 2006a)](https://paperpile.com/c/8sOC5X/Qh7L) and laboratory [(Langlois *et al.* 2006b)](https://paperpile.com/c/8sOC5X/K0G5), supporting the results of this analysis.

NTR status, distance and organic content were found to be important across all possible models explaining the abundance of *Myadora striata* (Fig. A4.2), and had strong model support according to AICc (Table A4.2). However a simpler model of decreasing abundance of *M. striata* with increasing density of legal-sized rock lobster was the most parsimonious model within 2AICc (Fig. A4.3), and had relatively high model support based on BIC (Table A4.2). A direct relationship with the density of legal-sized rock lobster is consistent with the observation that greater than legal-size rock lobster can readily prey upon bivalves [(Langlois *et al.* 2006b)](https://paperpile.com/c/8sOC5X/K0G5).

**Table A4.2.** Top generalised additive mixed models (GAMMs) for predicting the abundance of the bivalves *Donsinia subrosea* and *Myadora striata* and the hermit crab *Pagurus novizelandiae* from full subset analyses. Difference between lowest reported corrected Akaike Information Criterion (ΔAICc) and Bayesian Information Criterion (ΔBIC), both AICc and BIC weights (ωAICc and ωBIC), variance explained (R^2^) and effective degrees of freedom (EDF) are reported for model comparison.

|  | Best models | ΔAICc | ΔBIC | ωAICc | ωBIC | R^2^ | EDF |
| --- | --- | --- | --- | --- | --- | --- | --- |
| Bivalve |  |  |  |  |  |  |  |
| *Dosinia subrosea* | distance * Status + 500um + Status | 0 | 0 | 0.34 | 0.41 | 0.52 | 20.7 |
|  | distance * Status + fetch + Status | 0.23 | 0.57 | 0.30 | 0.31 | 0.46 | 21.2 |
|  | distance * Status + 500um * Status + Status | 1.26 | 1.73 | 0.18 | 0.17 | 0.51 | 21.1 |
| *Myadora striata* | distance * Status + organic * Status + Status | 0 | 5.09 | 0.71 | 0.07 | 0.54 | 23.3 |
|  | lobster | 2.00 | 0 | 0.26 | 0.86 | 0.44 | 16.5 |
| Hermit crab |  |  |  |  |  |  |  |
| *Pagurus novizelandiae* | 4mm + lobster | 0 | 0.47 | 0.05 | 0.05 | 0.48 | 19.5 |
|  | 4mm + 500um + lobster | 0.01 | 0.81 | 0.05 | 0.04 | 0.46 | 19.8 |
|  | 4mm + 500um | 0.22 | 1.04 | 0.05 | 0.04 | 0.47 | 19.9 |
|  | 4mm + 500um + snapper | 0.27 | 1.12 | 0.05 | 0.04 | 0.46 | 19.9 |
|  | 4mm + snapper | 0.28 | 0.82 | 0.05 | 0.04 | 0.47 | 19.6 |
|  | 4mm | 0.29 | 0.83 | 0.05 | 0.04 | 0.48 | 19.7 |
|  | 4mm + 500um + Status | 0.56 | 1.56 | 0.04 | 0.03 | 0.47 | 20.1 |
|  | 4mm+fetch+lobster | 0.59 | 1.25 | 0.04 | 0.03 | 0.48 | 19.7 |
|  | 4mm+Status | 0.63 | 1.34 | 0.04 | 0.03 | 0.48 | 19.8 |
|  | 4mm+fetch | 0.72 | 1.46 | 0.04 | 0.03 | 0.47 | 19.8 |
|  | 4mm+fetch+snapper | 0.91 | 1.73 | 0.03 | 0.03 | 0.47 | 19.8 |
|  | 4mm+fetch+Status | 1.11 | 2.0 | 0.03 | 0.02 | 0.47 | 19.9 |
|  | 1mm+lobster | 1.56 | 0 | 0.02 | 0.06 | 0.42 | 17.7 |
|  | 4mm+Status+fetch x Status | 1.58 | 2.9 | 0.02 | 0.02 | 0.47 | 20.4 |
|  | 4mm+org+snapper | 1.77 | 4.31 | 0.02 | 0.01 | 0.48 | 21.3 |
|  | 1mm+Status+Distance x Status | 1.84 | 3.04 | 0.02 | 0.01 | 0.46 | 20.2 |
|  | 1mm | 1.95 | 0.45 | 0.02 | 0.05 | 0.43 | 17.8 |
|  | 4mm+fetch+org | 1.96 | 4.62 | 0.02 | 0.01 | 0.49 | 21.5 |

| 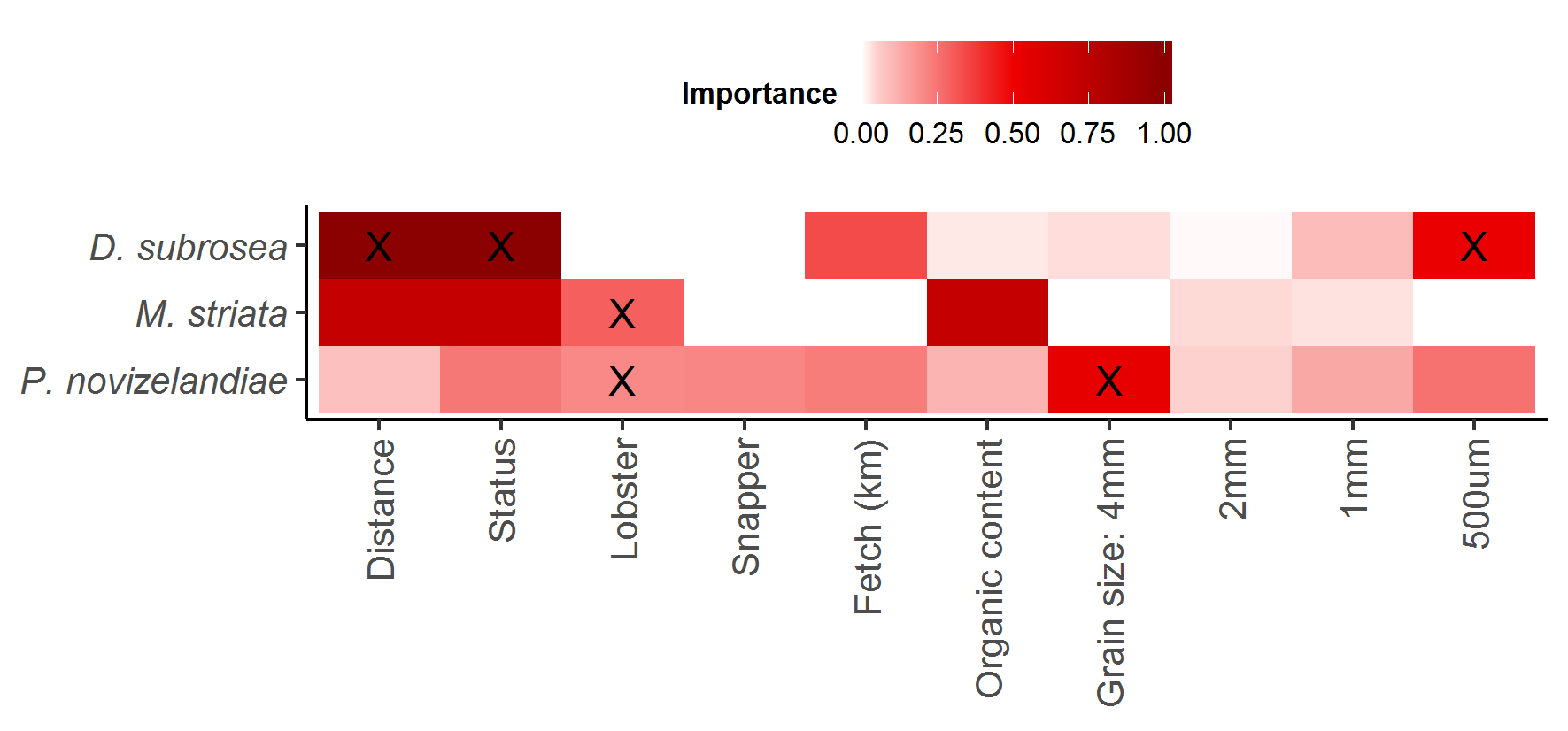 | **Fig. A4.2.** Variable importance scores from full subsets GAMM analyses to predict the abundance of the bivalves *Dosinia subrosea* and *Myadora striata* and the hermit crab *Pagurus novizelandiae*, variables within the most parsimonious model for each taxa are indicated (X, see Table 2.2). |
| --- | --- |

| *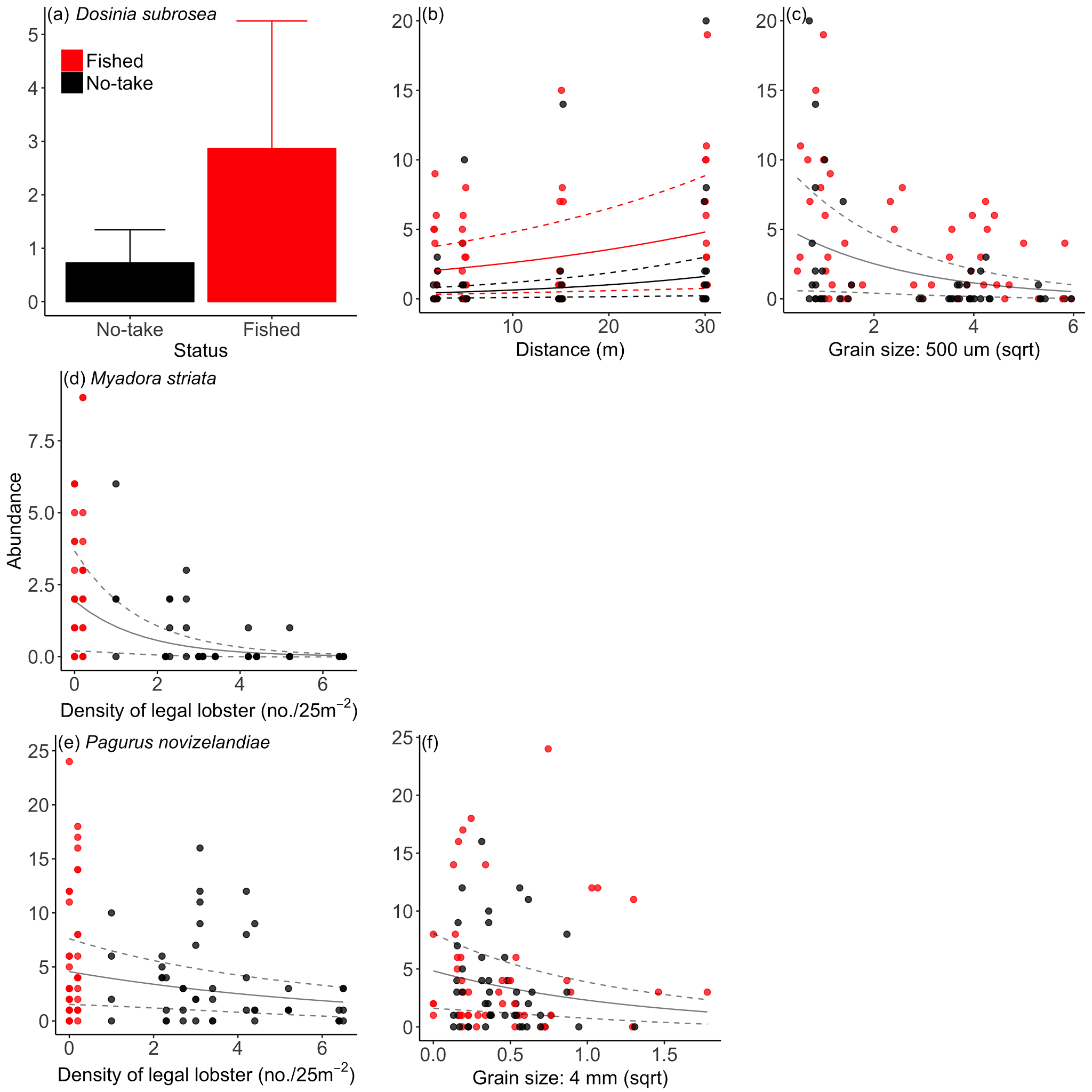* | | |
| --- | --- | --- |
| *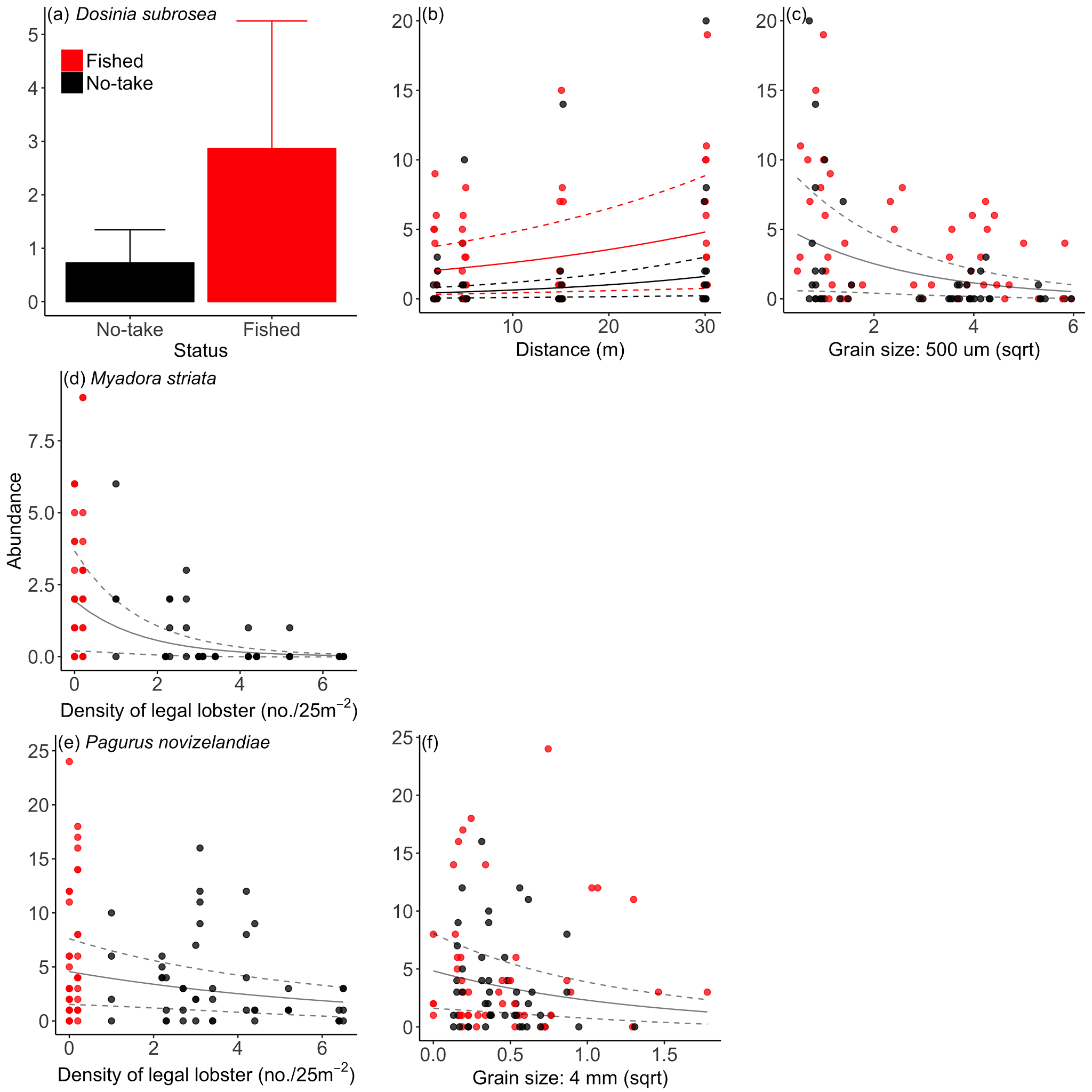* |  | **Fig A4.3.** Plots of most parsimonious model found to predict the abundance of the bivalves *Dosinia subrosea* and *Myadora striata* and the hermit crab *Pagurus novizelandiae* from full subset GAMM analyses (see Table A4.2). Solid lines are fitted gam curves, with dashed lines indicate standard error confidence bands. |
| *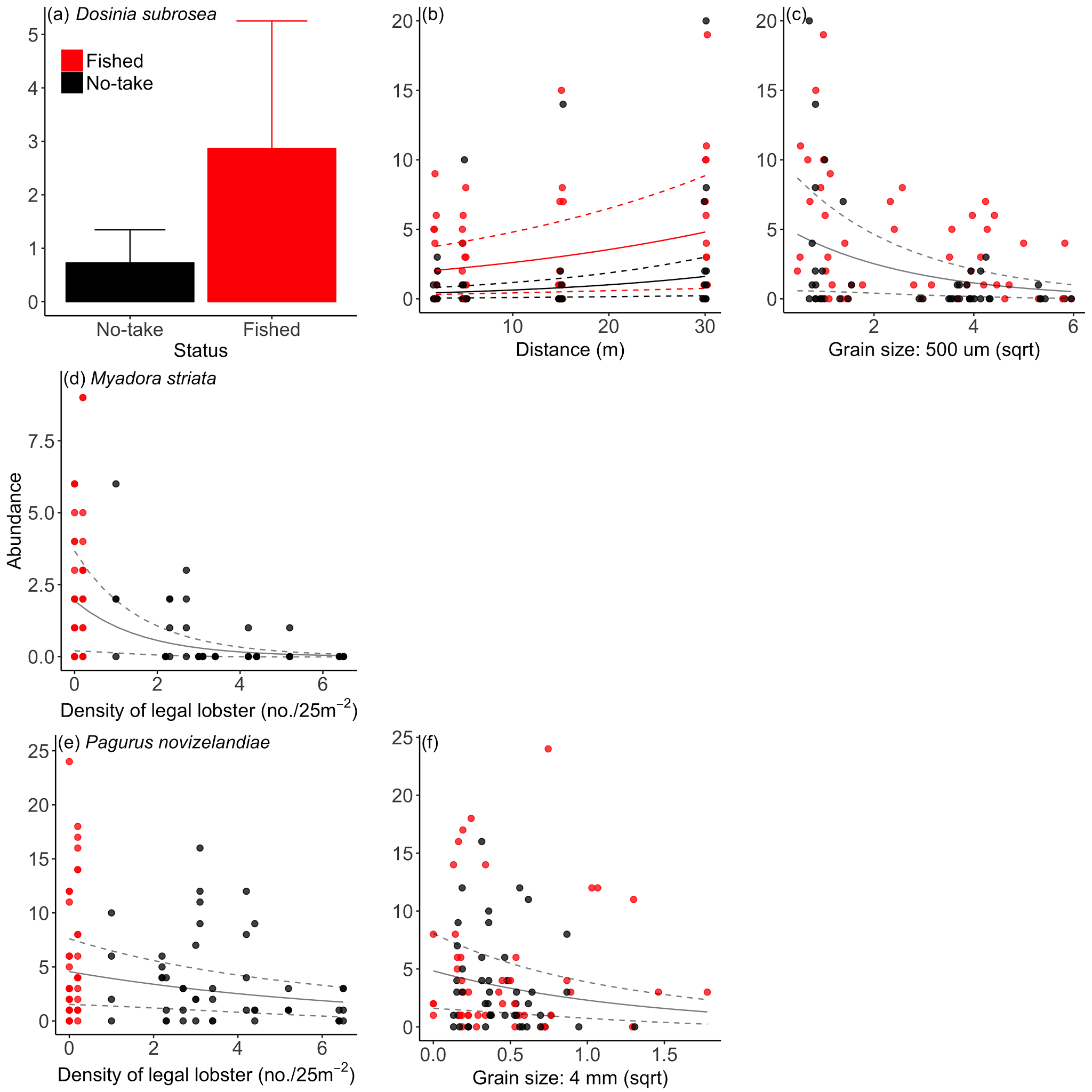* | |  |

There was a high level of model uncertainty in our full subsets analysis of the ubiquitous hermit crab *Pagurus novizelandiae*, with very low model weights (maximum wi 0.05, Table A4.2) and low, relatively evenly distributed variable importance scores (Figure A4.2). This is consistent with the original study that found no effect of NTR status on the abundance of *Pagurus novizelandiae*. The best model included the 4 mm sediment grain size fraction and the density of legal-sized rock lobster, explaining 48% of the distribution of *P. novizelandiae* (Table A4.2). With the abundance of *P. novizelandiae* being negatively correlated with increasing proportion of the 4 mm sediment grain size fraction and negatively correlated with the density of rock lobster on the reef edge (Fig. A4.3). The direct relationship between the density of legal-sized rock lobster and the hermit crab *P. novizelandiae* is consistent with feeding studies of rock lobster, which indicated that they can exhibit a strong preference for decapod prey [(Dumas *et al.* 2013)](https://paperpile.com/c/8sOC5X/iER2).

Overall the general results of the revised analysis are comparable to those from the original study. However, by allowing environmental information to be included in the new analysis, whilst accounting for the nesting and random allocation of sites and NTR locations, has allowed a more informed comparison of the abundance of ubiquitous infaunal taxa. In particular, it is useful that instead of relying on a simple comparison of NTR status to test hypothesis regarding predation of infauna, the role of the large carnivorous species protected with the NTR (e.g. rock lobster) can be more clearly indicated once it is included as a predictor variable in the models.

## References

[Ambrose, W.G., Jr. (1991). Are infaunal predators important in structuring marine soft-bottom communities? *American Zoologist*, **31**, 849–860.](http://paperpile.com/b/8sOC5X/mRoe)

[Anderson, M.J. & ter Braak, C.J.F. (2003). Permutation tests for multi-factorial analysis of variance. *Journal of Statistical Computation and Simulation*, **73**, 85–113.](http://paperpile.com/b/8sOC5X/u2tj)

[Auguie, B. (2016). gridExtra: Miscellaneous Functions for ‘Grid’ Graphics. R package version 2.2.1. URL https://CRAN.R-project.org/package=gridExtra [accessed 22 June 2017].](http://paperpile.com/b/8sOC5X/Rd8f)

[Babcock, R.C., Kelly, S., Shears, N.T., Walker, J.W. & Willis, T.J. (1999). Changes in community structure in temperate marine reserves. *Marine Ecology Progress Series*, **189**, 125–134.](http://paperpile.com/b/8sOC5X/rMkD)

[Beck, M.W. (1997). Inference and generality in ecology - current problems and an experimental solution. *Oikos* , **78**, 265–273.](http://paperpile.com/b/8sOC5X/GeZr)

[Dahlgren, C.P., Posey, M.H. & Hulbert, A.W. (1999). The effects of bioturbation on the infaunal community adjacent to an offshore hardbottom reef. *Bulletin of Marine Science*, **64**, 21–34.](http://paperpile.com/b/8sOC5X/5tXL)

[Davis, N., Vanblaricom, G.R. & Dayton, P.K. (1982). Man-made structures on marine sediments effects on adjacent benthic communities. *Marine Biology*, **70**, 295–304.](http://paperpile.com/b/8sOC5X/AxVk)

[Dumas, J.-P., Langlois, T.J., Clarke, K.R. & Waddington, K.I. (2013). Strong preference for decapod prey by the western rock lobster Panulirus cygnus. *Journal of Experimental Marine Biology and Ecology*, **439**, 25–34.](http://paperpile.com/b/8sOC5X/iER2)

[Fairweather, P.G. (1988). Predation creates haloes of bare space among prey on rocky seashores in New South Wales. *Journal of Experimental Marine Biology and Ecology*, **114**, 261–273.](http://paperpile.com/b/8sOC5X/w8bM)

[Hurlbert, S.H. (1984). Pseudoreplication and the Design of Ecological Field Experiments. *Ecological Monographs*, **54**, 187–212.](http://paperpile.com/b/8sOC5X/J4MA)

[Kelly, S. (2001). Temporal variation in the movement of the spiny lobster Jasus edwardsii. *Marine and Freshwater Research*, **52**, 323–331.](http://paperpile.com/b/8sOC5X/rdw1)

[Kelly, S., MacDiarmid, A.B. & Babcock, R.C. (1999). Characteristics of spiny lobster, Jasus edwardsii, aggregations in exposed reef and sandy areas. *Marine and Freshwater Research*, **50**, 409–416.](http://paperpile.com/b/8sOC5X/yNlG)

[Langlois, T.J., Anderson, M.J. & Babcock, R.C. (2005). Reef-associated predators influence adjacent soft-sediment communities. *Ecology*, **86**, 1508–1519.](http://paperpile.com/b/8sOC5X/cQ8I)

[Langlois, T.J., Anderson, M.J., Babcock, R.C. & Kato, S. (2006a). Marine reserves demonstrate trophic interactions across habitats. *Oecologia*, **147**, 134–140.](http://paperpile.com/b/8sOC5X/Qh7L)

[Langlois, T.J., Anderson, M.J., Brock, M. & Murman, G. (2006b). Importance of rock lobster size-structure for trophic interactions: choice of soft-sediment bivalve prey. *Marine Biology*, **149**, 447–454.](http://paperpile.com/b/8sOC5X/K0G5)

[Lindquist, D.G., Cahoon, L.B., Clavijo, I.E., Posey, M.H., Bolden, S.K., Pike, L.A., Burk, S.W. & Cardullo, P.A. (1994). Reef fish stomach contents and prey abundance on reef and sand substrata associated with adjacent artificial and natural reefs in Onslow Bay, North Carolina. *Bulletin of Marine Science*, **55**, 1994.](http://paperpile.com/b/8sOC5X/dyDZ)

[MacDiarmid, A.B. (1991). Seasonal changes in depth distribution sex ratio and size frequency of spiny lobster (Jasus edwardsii) on a coastal reef in northern New Zealand. *Marine Ecology Progress Series*, **70**, 129–141.](http://paperpile.com/b/8sOC5X/C0C3)

[Parsons, D.M., Babcock Russ, C., Hankin, R.K.S., Willis Trevor, J., Aitken, J.P., O’Dor, R.K. & Jackson, G.D. (2003). Snapper Pagrus auratus (Sparidae) home range dynamics: acoustic tagging studies in a marine reserve. *Marine Ecology Progress Series*, **262**, 253–265.](http://paperpile.com/b/8sOC5X/aI5v)

[Posey, M.H. & Ambrose, W.G. (1994). Effects of proximity to an offshore hard-bottom reef on infaunal abundances. *Marine Biology*, **118**, 745–753.](http://paperpile.com/b/8sOC5X/R46L)

[R Core Team. (2017). R: A Language and Environment for Statistical Computing. R Foundation for Statistical Computing Vienna, Austria. URL https://www.R-project.org/ [accessed 22 June 2017].](http://paperpile.com/b/8sOC5X/sIOd4)

[Shears, N.T. & Babcock, R.C. (2002). Marine reserves demonstrate top-down control of community structure on temperate reefs. *Oecologia*, **132**, 131–142.](http://paperpile.com/b/8sOC5X/Ehv5)

[Shears, N.T. & Babcock, R.C. (2003). *Quantitative classification of New Zealand rocky coastal community types*. Department of Conservation, Wellington, New Zealand.](http://paperpile.com/b/8sOC5X/5ZVp)

[Suchanek, T.H. (1978). The ecology of Mytilus edulis in exposed rocky inter tidal communities. *Journal of Experimental Marine Biology and Ecology*, **31**, 105–120.](http://paperpile.com/b/8sOC5X/RLkE)

[Thomas, M.L.H. (1986). A physically derived exposure index for marine shorelines. *Ophelia*, **25**, 1–13.](http://paperpile.com/b/8sOC5X/fdId)

[Thrush, S.F., Hewitt, J.E., Cummings, V.J., Green, M.O., Funnell, G.A. & Wilkinson, M.R. (2000). The generality of field experiments: interactions between local and broad-scale processes. *Ecology*, **81**, 399–415.](http://paperpile.com/b/8sOC5X/pJGn)

Underwood, A.J. (1993). The mechanics of spatially replicated sampling programmes to detect environmental impacts in a variable world. Austral Ecology, **18**, 99-116.

[Underwood, A.J., Chapman, M.G. & Connell, S.D. (2000). Observations in ecology: you can’t make progress on processes without understanding the patterns. *Journal of Experimental Marine Biology and Ecology*, **250**, 97–115.](http://paperpile.com/b/8sOC5X/icAl)

[Wickham, H. (2009). *ggplot2: Elegant Graphics for Data Analysis*. Springer-Verlag New York.](http://paperpile.com/b/8sOC5X/4T9E)

[Wickham, H. (2017). tidyr: Easily Tidy Data with ‘spread()’ and ‘gather()’ Functions. R package version 0.6.1. URL https://CRAN.R-project.org/package=tidyr [accessed 22 June 2017].](http://paperpile.com/b/8sOC5X/zE4a)

[Wickham, H. & Francois, R. (2016). dplyr: A Grammar of Data Manipulation. R package version 0.5.0. URL https://CRAN.R-project.org/package=dplyr [accessed 22 June 2017].](http://paperpile.com/b/8sOC5X/H4ZZ)

[Willis, T.J., Millar, R.B. & Babcock, R.C. (2003). Protection of exploited fish in temperate regions: high density and biomass of snapper Pagrus auratus (Sparidae) in northern New Zealand marine reserves. *The Journal of Applied Ecology*, **40**, 214–227.](http://paperpile.com/b/8sOC5X/3mkh)

[Willis Trevor, J. & Babcock Russell, C. (2000). A baited underwater video system for the determination of relative density of carnivorous reef fish. *Marine & Freshwater Research*, **51**, 755–763.](http://paperpile.com/b/8sOC5X/lUAk)

[Wood, S.N. (2011). Fast stable restricted maximum likelihood and marginal likelihood estimation of semiparametric generalized linear models. *Journal of the Royal Statistical Society. Series B, Statistical methodology*, **73**, 3–36.](http://paperpile.com/b/8sOC5X/7HYk)

[Wood, S.N. (2017). *Generalized Additive Models: An Introduction with R, Second Edition*. CRC Press.](http://paperpile.com/b/8sOC5X/8Bn0)
