# Supplementary material for: A simple function for full‐subsets multiple regression in ecology with R
Source: Ecol Evol. 2018 May 20;8(12):6104–13. doi: 10.1002/ece3.4134 (PMC6024142; doi:10.1002/ece3.4134)
Supplement: Supplementary file 5 [file ECE3-8-6104-s005.docx]

# Case Study 3: Reproductive patterns of tropical intertidal invertebrates over multiple temporal scales

## Background

Studies of reproductive biology are fundamental to understanding resource allocation, larval recruitment and population dynamics [(Underwood & Keough 2001)](https://paperpile.com/c/SLRoeo/SZWW). Moreover, these studies provide valuable insights into life history strategies, uncovering important interactions with environmental conditions and habitats, therefore supporting the development of appropriate measures for conservation and management. In addition, for species of commercial importance, these studies also provide in situ parameters critical for population modelling and prediction, underpinning efforts to ensure sustainable fishing.

Reproductive cycles can occur at a number of scales, ranging (in decreasing frequency) from circadian, semi-lunar and lunar to seasonal. Despite an apparent lack of pronounced seasonality in climate in the tropics, many organisms display seasonal peaks in reproductive activity [(Brown & Shine 2006)](https://paperpile.com/c/SLRoeo/uskN), the most striking example being that of annual or bi-annual synchronous spawning in scleractinian corals [(e.g. Babcock *et al.* 1994)](https://paperpile.com/c/SLRoeo/Fam4/?prefix=e.g.). In marine systems, lunar and semi-lunar cycles are an obvious cue for reproduction, particularly for broadcast spawners, for which synchronicity is critical for fertilization success [(Babcock *et al.* 1992)](https://paperpile.com/c/SLRoeo/nFa0). Few studies have concurrently examined effects of annual and lunar patterns on the spawning of marine invertebrates in the tropics in a manner that elucidates relative reproductive output at both scales. Many of these studies address issues of annual spawning period (in terms of months) and only makes mention that reproductive activity appeared higher during spring/neap tides during those spawning months but did not address within-month variation specifically. This can potentially confound data related to reproductive biorhythmicity, particularly for intertidal organisms, which are collected on a semi-regular basis based on tide height, which inherently coincides with lunarity. This can result in confusion over the conclusions made about the reproductive patterns and cycles, where sampling timing often becomes a confounding factor.

Part of the issue may relate to the fact that both lunar and seasonal patterns are cyclical in nature, representing a challenge in conventional/traditional analyses. Calendar month is frequently denoted as a categorical factor and statistically evaluated via Analyses of Variance (ANOVA) or similar techniques [(e.g. Liu 1994; Ettinger-Epstein *et al.* 2007)](https://paperpile.com/c/SLRoeo/IKr5+Brrr/?prefix=e.g.,). Formal elucidation of lunar and semilunar cycles mostly take two major forms in the literature: (a) factorial analyses in which moon phase is distributed into two to four categories [(e.g. Iliffe & Pearse 1982)](https://paperpile.com/c/SLRoeo/mAZX/?prefix=e.g.); and (b) periodic regression where analyses are conducted on sine and cosine angular terms that prescribe lunar days [(e.g. Cavraro *et al.* 2014)](https://paperpile.com/c/SLRoeo/x8Hs/?prefix=e.g.). Categorized moon phase is usually analysed via ANOVA, although alternative methods applied include Chi-squared contingency tests [(Battaglene *et al.* 2002)](https://paperpile.com/c/SLRoeo/k7Og), and paired t-tests (within months) have also been applied to two-level categorization of moon phases [(e.g. Iliffe & Pearse 1982)](https://paperpile.com/c/SLRoeo/mAZX/?prefix=e.g.). Periodic regression [(see deBruyn & Meeuwig 2001)](https://paperpile.com/c/SLRoeo/qbCm/?prefix=see) takes the form of equation 1 and is beneficial in that it correctly captures the cyclical nature of lunar days, and not constraining this temporal element to categories allows for regressive approaches.

y = b_0_ + b_1_ sin θ +b_2_ cos θ + b_3_ sin 2θ + b_4_ cos 2θ, Eqn 1

where y is the dependent variable, θ is lunar day in radians, and b_0_- b_4_ are estimated from the regression. The parameter estimates for lunar day are coefficients which define phase shift and amplitude [(Batschelet 1981)](https://paperpile.com/c/SLRoeo/cE7I). The terms, sin 2θ and cos 2θ, were used to detect semi-lunar cycles [(deBruyn & Meeuwig 2001)](https://paperpile.com/c/SLRoeo/qbCm). The downside of applying either categorical ANOVAs or periodic regression to the elucidation of biorhythmic cycles in reproduction is the number of parameters that implicitly must be estimated, i.e. one per level of factor, and five for the periodic regression, although a model-building approach could lead to the exclusion of semi-lunar cycles, if appropriate.

In this case study, General Additive Models (GAMs) were used to elucidate reproductive patterns at multiple temporal scales in two species of broadcast spawning gastropods. GAMs were first proposed by Hastie and Tibshirani [(1990)](https://paperpile.com/c/SLRoeo/OAG0/?noauthor=1), and are able to deal with non-linear relationships between an independent variable and multiple, potentially interactive predictors in the same model. GAMs have been widely used to model intra-annual trends in the abundances [(e.g. Bellido *et al.* 2001)](https://paperpile.com/c/SLRoeo/Tyo2/?prefix=e.g.), condition and reproductive activity [(Dunn & Forman 2011)](https://paperpile.com/c/SLRoeo/mcqa) of commercially important species, but remain relatively rarely applied to non-fishery species [(but see Guijarro *et al.* 2012)](https://paperpile.com/c/SLRoeo/a8De/?prefix=but%20see). The full subsets gam function also allows other factors (e.g. sex) to be examined as both interactions (e.g. a different relationship with lunar day within each level), and as main effects (ie a shift in the overall relationship up or down within each level). *Patelloida saccharina* and *Monodonta labio* are common in the rocky intertidal, and are among the most abundant gastropods inhabiting artificial seawalls and breakwaters in Singapore [(Lee *et al.* 2009)](https://paperpile.com/c/SLRoeo/PXri). To examine this, the reproductive cycles of these two gastropods, determined by gonadosomatic index, were assessed across three temporal scales; among years, among months (i.e. yearly pattern), and within month (i.e. lunar and semi-lunar cycle).

## Methods

Samples were collected from St. John’s Island (southern island complex), Singapore (1° 16.8’N, 103° 52.2’E). Reproductive cycles for *Patelloida saccharina* and *Monodonta labio* was determined from 10 – 15 individuals (shell length 7 – 25 mm for *P. saccharina* and 10 – 30 mm for *M. labio*) that were collected from July 2003 to July 2004. To examine the possible effect of lunar periodicity on the reproductive cycles, were collected every three days (where possible) in the first five months (July 2003 till November 2003). Three day sampling was conducted to establish patterns of reproduction and to eliminate a foregone conclusion of semi-lunar or lunar effects. Initial 3-day sampling indicated a fairly consistent cyclical pattern in GSI for both *M. labio* and *P. saccharina* that appeared associated with the lunarity. Based on this finding, sampling frequency was reduced to every 7 days for the following 8 months.

Specimens were relaxed in 1:1 7.5% MgCl: seawater solution and then preserved in 4% buffered seawater formalin. After preservation, individuals were removed from their shells and gonadal material removed and the wet weight of gonadal and somatic tissues were obtained. Gonadosomatic Index (GSI=wet weight gonad/wet weight somatic × 100). Histology sections were also prepared for female gonads to validate the use of GSI as a measure of reproductive output. Sections were stained with Mayer’s Haemotoxylin and Eosin and mounted in DPX mountant.

Quantification of oocytes was conducted in triplicate 500 μm quadrats. Staging of the oocytes were based in part on previous studies conducted by Orton *et al.* [(1956)](https://paperpile.com/c/SLRoeo/v6CC/?noauthor=1), Underwood [(1974)](https://paperpile.com/c/SLRoeo/h0rR/?noauthor=1) and Liu [(1994)](https://paperpile.com/c/SLRoeo/IKr5/?noauthor=1). Primordial and immature (Stage 1) are small (4-19 μm for *Patelloida saccharina* and 10-20 μm for *Monodonta labio*) and attached to the follicle wall. A dark staining nucleus is visible at this stage (Fig A5.1a, c). Maturing oocytes (stage 2) are larger than Stage 1 oocytes (14-46 μm for *P. saccharina* and 45-92 μm for *M. labio*), still attached to the follicle wall and with distinct nucleus and nucleolus (Fig A5.1a, c). Stage 3 or mature oocytes are large, 55-125 μm and 97-125 μm for *P. saccharina* and *M. labio* respectively, irregularly shaped and densely packed with yolk granules. The nucleus becomes less apparent but the nucleolus is large and is still present (Fig A5.1b, d).

A total of 744 individuals of *Patelloida saccharina* and 549 individuals of *Monodonta labio* were collected. Histological examination showed a strong relationship between GSI and maturity of gonad in *Patelloida saccharina*, making it a good proxy for measures of reproductive levels. The gonad primarily consisted of high proportions of Stage 1 oocytes (> 40% of total number of oocytes) and low numbers of Stage 3 oocytes (0-40%) at low GSI. High GSI values are represented by higher numbers of Stage 3 oocytes, which make up at least 60% of total number of oocytes. There were also more numbers of Stage 2 oocytes than Stage 1 at high GSI values.

The relationship between gonad maturity and GSI was less apparent in *Monodonta labio* than it was in *P. saccharina*. Ripe oocytes were evident in *M. labio* throughout the study period with some indication of increased intensity at certain times of the year, usually corresponding with increased GSI. There was relatively high proportion of Stage 3 oocytes (between 20-60%) even at low GSI. However, at these GSI levels, higher proportions Stage 1 oocytes than Stage 2 were observed. High GSI values in *M. labio* are represented by higher number of Stage 3 oocytes, moderate numbers of Stage 2 oocytes (between 30-40%) and relatively lower proportion of Stage 1 oocytes, which only make up 10-20% of total number of oocytes. Although the pattern is less apparent, the levels of GSI are nonetheless an appropriate proxy for measures of reproductive levels in *M. labio*.


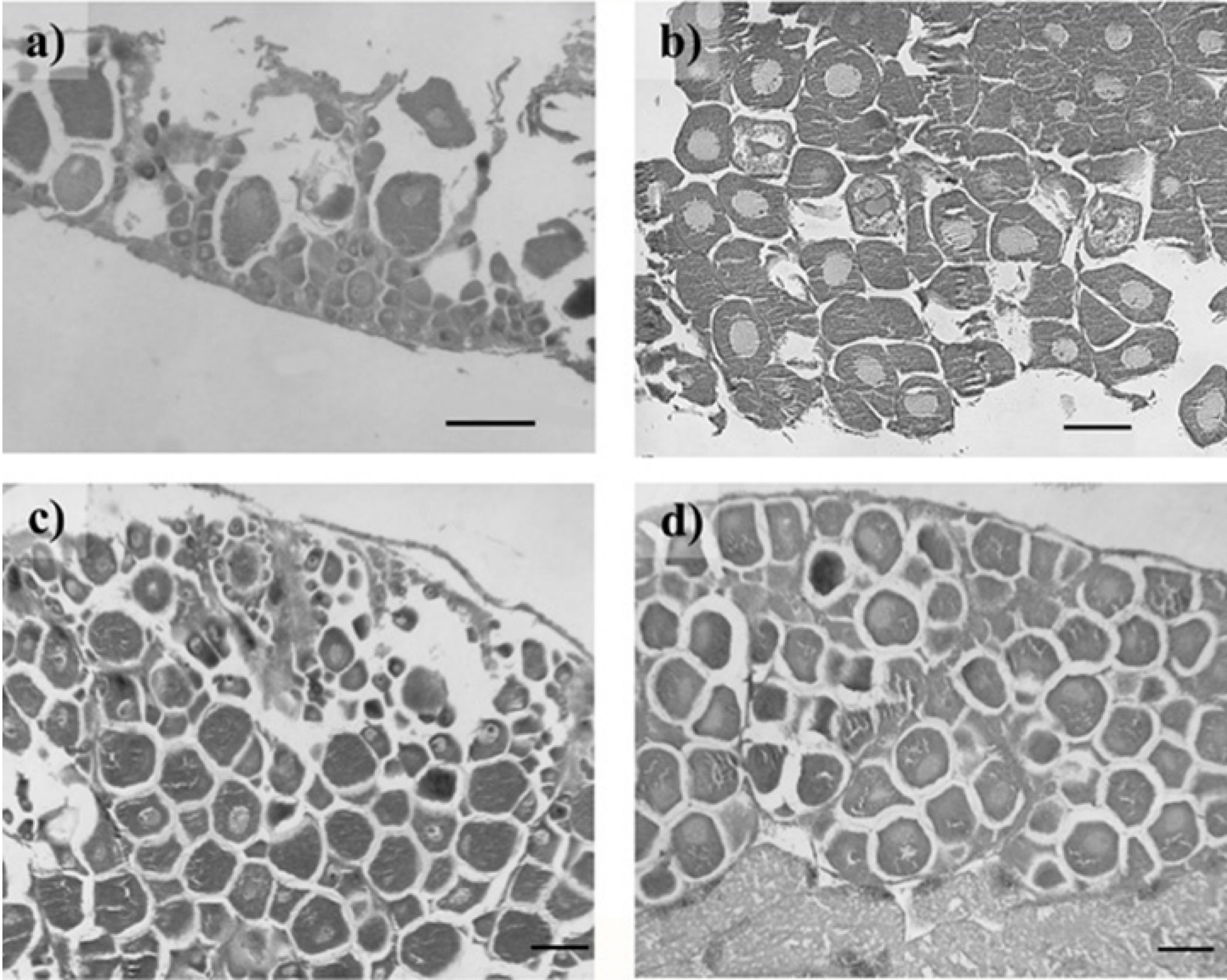


**Fig. A5.1.** Proportion of oocyte stages in a) *Patelloida saccharina* and b) *Monodonta labio*. Stages enumerated in a 500 μm X 500 μm quadrat

### Data analyses

We used a generalized additive modelling approach to examine factors influencing GSI in limpets at St John’s Island in Singapore. Models were fit using gam, with GSI (the response variable in this case study) modelled using a gamma distribution, as high variable is a continuous response that was slightly skewed and does not contain zero. Two continuous predictors were included: month (1:12, capturing within year periodicity); and lunar date (1:30, capturing within month lunar, and semi-lunar periodicity). Both were modelled with cyclic cubic regression splines [(a penalized cubic regression splines whose ends match, up to second derivative, Wood 2006)](https://paperpile.com/c/SLRoeo/1rGL/?prefix=a%20penalized%20cubic%20regression%20splines%20whose%20ends%20match%2C%20up%20to%20second%20derivative%2C) by supplying the argument cyclic.vars. Species (*Monodonta labio* and *Patelloida saccharina)* and sex (female and male) were included as factors. All continuous predictors and factors were considered using the full subsets gam function. This was achieved via the full.susbets.gam function by leaving the factor.smooth.interactions argument on the default setting (which includes all factors as interaction terms with the continuous terms by default), and setting the factor.factor.interactions argument to “T”. This would allow all interactions between the factors to be considered, with potentially different lunar or monthly patterns for each sex and species. Interpretation of an interaction between Species and lunar date and month is straightforward (different species may have different peak spawning periods). While it would have been possible to exclude interactions between sex and lunar date and month on the assumption that peak spawning periods should be the same for both sexes (this is achieved by passing “Species” to the factor.smooth.interactions argument, which would limit the “by” argument interactions only to Species) we decided in this case to allow interactions with sex to be included, to explore the possibility that the magnitude of difference between the sexes changes with temporally. We also explored possible interactions between lunar date and month by setting the argument smooth.smooth.interactions to T. As this case study had only two continuous predictors, all factors had few levels, and the sample size was relatively high (n=1110) we decided to include models up to a maximum of 4 predictors (max.predictors=4). Correlations among predictors were very low for this case study (highest estimated correlation ~0.05), thus all models were retained in the candidate model set. All R code and the dataset used in analysis can be found at:

<https://github.com/beckyfisher/FSSgam/blob/master/case_study3_reproductive_cycles.R>

## Results & Discussion

A model with both lunar date and month as interactions with species, along with an intercept effect of sex, showed the highest ranking according to both AICc and BIC, explaining 28% of the variance in GSI for these species (Table A5.1). Plots of this top model indicated that the strong interactions observed for lunar day and month were due to markedly different trends for each of these predictors across the two species. A strong semi-lunar pattern was detected in *P. saccharina*, with fairly equal peaks in GSI occurring around lunar days 7 and 23 (Fig A5.2). Minima in GSI occurred around days 0 and 15 (Fig A5.2). This pattern correlates with minimal tidal range over the sampling period. Lunar patterns were generally much weaker for *M. labio* GSI, and were also reversed to that observed in *P. saccharina* (Fig A5.2)*.* Peaks were centered on lunar days 0 and 15, with bimodal minima in GSI occurring around days 5 and 23 (Fig A5.2). The magnitude of lunar periodicity on GSI was also markedly less pronounced in *M. labio* than in *P. saccharina*, in which lunar effects were several times that of seasonal effects.

**Table A5.1.** Top six model fits for full subsets analysis of gonadosomatic index (GSI) data. Shown are delta AICc and BIC, AICc and BIC weights, estimated R2 value and the total model estimated degrees of freedom.

| Model | ΔAICc | ΔBIC | ωAICc | ωBIC | R^2^ | Total edf |
| --- | --- | --- | --- | --- | --- | --- |
| Sex + Species + lunar date × Species + month × Species | 0.0 | 0.0 | 0.998 | 1 | 0.28 | 14.0 |
| Sex × Species + lunar date × Sex × Species + month × Sex × Species | 12.4 | 53.4 | 0.002 | 0 | 0.27 | 23.3 |
| Species + lunar date × Species + month × Species | 64.1 | 58.7 | 0 | 0 | 0.23 | 12.9 |
| month + Sex + Species + lunar date × Species | 106.8 | 93.2 | 0 | 0 | 0.21 | 11.2 |
| Sex + Species + month × Sex + lunar date × Species | 111.5 | 109.2 | 0 | 0 | 0.21 | 13.5 |
| Lunar date + Sex + Species + month × Species | 111.5 | 98.1 | 0 | 0 | 0.22 | 11.3 |


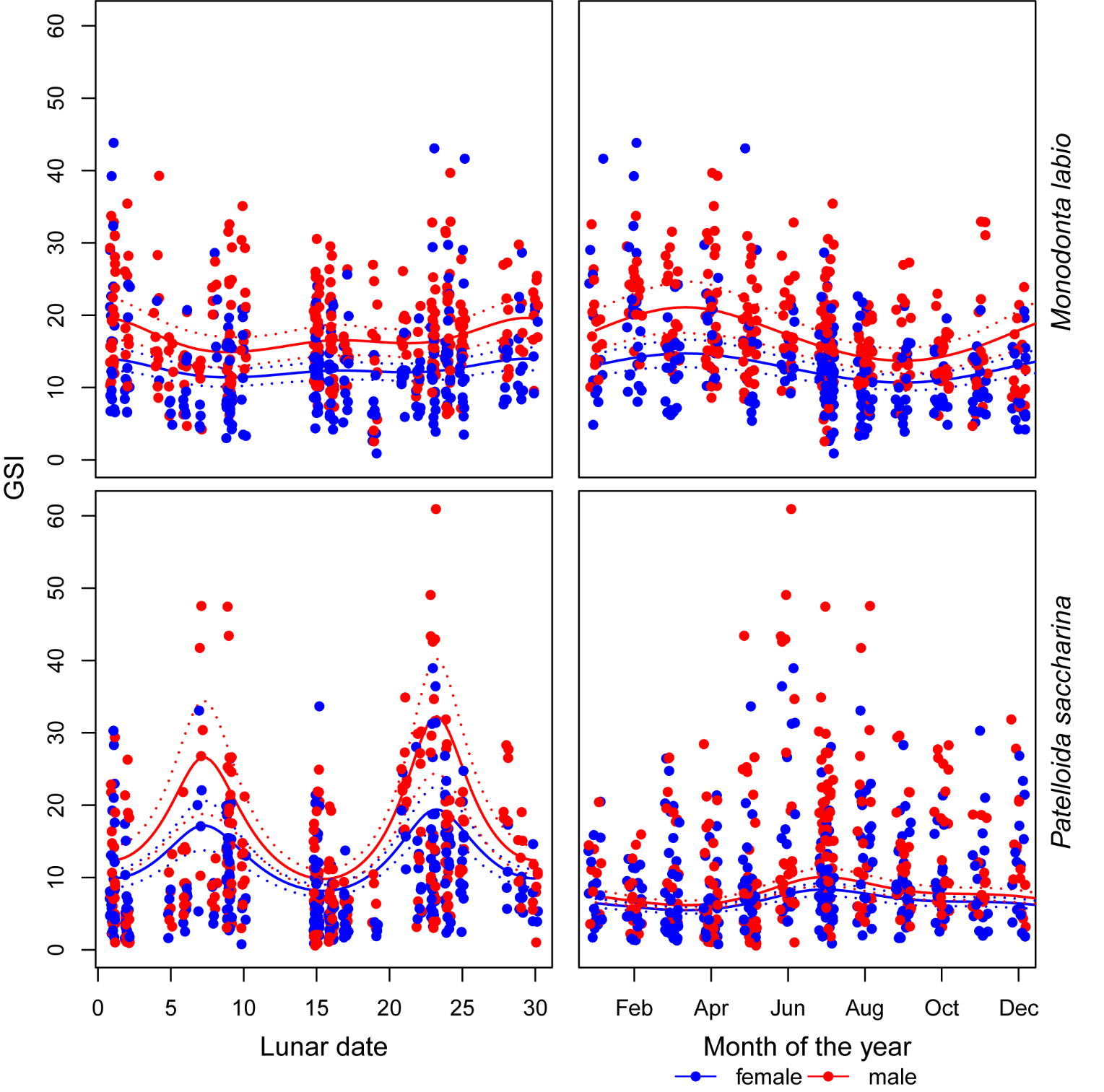


**Fig A5.2.** Visualization of the most parsimonious model for predicting gonadosomatic index (GSI) as ranked by AICc and BIC. Raw data are plotted separately for *Monodonta labio* *and Patelloida saccharina*, as a function of lunar date (left hand plots) and month of the year (right hand plots). Colors indicate sex (male and female). Solid lines are fitted gam curves, with dashed lines indicate 95% confidence bands.

Initial examinations suggest that both *Patelloida saccharina* and *Monodonta labio* are continuous breeders, indicated by GSI values that do not drop below five throughout the year. Near continuous breeding or extended breeding, with slight peaks in intensity during specific times/seasons have previously been reported in trochids [(see Hickman 1992 for review)](https://paperpile.com/c/SLRoeo/Ndpd/?prefix=see&suffix=for%20review) and acmaeids [(Creese 1980; Catalan & Yamamoto 1993)](https://paperpile.com/c/SLRoeo/TwQn+LklC). However, there were differences in the timing of peak reproduction between the two species throughout the year, with *M. labio* showing the highest output during February and March, and *P. saccharina* showing peak output in July (Fig A5.2). February/March denote the end of the Northeast monsoon, with increasing seawater temperatures over the following months, reaching annual peaks around August [(Sin *et al.* 2016)](https://paperpile.com/c/SLRoeo/4byK). Changes in temperature are known proximate indicators reproductive seasonality in both limpets as well as trochaceans [(Morton & Morton 1983)](https://paperpile.com/c/SLRoeo/kcZG). However, additional factors such as the seasonal availability of food availability resources, may also contribute to the trigger of gametogenesis [(Hickman 1992)](https://paperpile.com/c/SLRoeo/Ndpd), but were not examined in this study. Values of GSI were higher in males compared to females for both species, regardless of lunar day, or time of year (Fig A5.2). This is a common phenomenon in intertidal gastropods [(Creese 1980; Creese & Ballantine 1983; Liu 1994)](https://paperpile.com/c/SLRoeo/TwQn+hDYd+IKr5).

## References

[Babcock, R., Mundy, C., Keesing, J. & Oliver, J. (1992). Predictable and unpredictable spawning events: in situ behavioural data from free-spawning coral reef invertebrates. *Invertebrate Reproduction & Development*, **22**, 213–227.](http://paperpile.com/b/SLRoeo/nFa0)

[Babcock, R.C., Wills, B.L. & Simpson, C.J. (1994). Mass spawning of corals on a high latitude coral reef. *Coral Reefs* , **13**, 161–169.](http://paperpile.com/b/SLRoeo/Fam4)

[Batschelet, E. (1981). *Circular Statistics in Biology*. Academic Press.](http://paperpile.com/b/SLRoeo/cE7I)

[Battaglene, S.C., Seymour, J.E., Ramofafia, C. & Lane, I. (2002). Spawning induction of three tropical sea cucumbers, Holothuria scabra, H. fuscogilva and Actinopyga mauritiana. *Aquaculture* , **207**, 29–47.](http://paperpile.com/b/SLRoeo/k7Og)

[Bellido, J.M., Pierce, G.J. & Wang, J. (2001). Modelling intra-annual variation in abundance of squid Loligo forbesi in Scottish waters using generalised additive models. *Fisheries Research*, **52**, 23–39.](http://paperpile.com/b/SLRoeo/Tyo2)

[Brown, G.P. & Shine, R. (2006). Why do most tropical animals reproduce seasonally? Testing hypotheses on an Australian snake. *Ecology*, **87**, 133–143.](http://paperpile.com/b/SLRoeo/uskN)

[Catalan, M.A.A.B. & Yamamoto, M. (1993). Annual reproductive cycle of the prosobranch limpet, Cellana nigrolineata (Reeves). *Invertebrate Reproduction & Development*, **24**, 127–136.](http://paperpile.com/b/SLRoeo/LklC)

[Cavraro, F., Varin, C. & Malavasi, S. (2014). Lunar-induced reproductive patterns in transitional habitats: Insights from a Mediterranean killifish inhabiting northern Adriatic saltmarshes. *Estuarine, Coastal and Shelf Science*, **139**, 60–66.](http://paperpile.com/b/SLRoeo/x8Hs)

[Creese, R.G. (1980). Reproductive cycles and fecundities of four common eastern Australian archaeogastropod limpets (Mollusca: Gastropoda). *Marine and Freshwater Research*.](http://paperpile.com/b/SLRoeo/TwQn)

[Creese, R.G. & Ballantine, W.J. (1983). An assessment of breeding in the intertidal limpet, Cellana radians (Gmelin). *Journal of Experimental Marine Biology and Ecology*, **67**, 43–59.](http://paperpile.com/b/SLRoeo/hDYd)

[deBruyn, A.M.H. & Meeuwig, J.J. (2001). Detecting lunar cycles in marine ecology: periodic regression versus categorical ANOVA. *Marine Ecology Progress Series*, **214**, 307–310.](http://paperpile.com/b/SLRoeo/qbCm)

[Dunn, M.R. & Forman, J.S. (2011). Hypotheses of Spatial Stock Structure in Orange Roughy Hoplostethus atlanticus Inferred from Diet, Feeding, Condition, and Reproductive Activity. *PloS one*, **6**, e26704.](http://paperpile.com/b/SLRoeo/mcqa)

[Ettinger-Epstein, P., Whalan, S.W., Battershill, C.N. & de Nys, R. (2007). Temperature cues gametogenesis and larval release in a tropical sponge. *Marine Biology*, **153**, 171–178.](http://paperpile.com/b/SLRoeo/Brrr)

[Guijarro, B., Fanelli, E., Moranta, J., Cartes, J.E. & Massutí, E. (2012). Small-scale differences in the distribution and population dynamics of pandalid shrimps in the western Mediterranean in relation to environmental factors. *Fisheries Research*, **119**, 33–47.](http://paperpile.com/b/SLRoeo/a8De)

[Hastie, T.J. & Tibshirani, R.J. (1990). *Generalized Additive Models*. CRC Press.](http://paperpile.com/b/SLRoeo/OAG0)

[Hickman, C.S. (1992). Reproduction and development of trochacean gastropods. *The Veliger*, **35**, 245–272.](http://paperpile.com/b/SLRoeo/Ndpd)

[Iliffe, T.M. & Pearse, J.S. (1982). Annual and lunar reproductive rhythms of the sea urchin, Diadema antillarum (Philippi) in Bermuda. *International Journal of Invertebrate Reproduction*, **5**, 139–148.](http://paperpile.com/b/SLRoeo/mAZX)

[Lee, A.C., Sin, T.M. & Others. (2009). Intertidal assemblages on coastal defence structures in Singapore II: Contrasts between islands and the mainland. *The Raffles Bulletin of Zoology*, **22**, 255–268.](http://paperpile.com/b/SLRoeo/PXri)

[Liu, J.H. (1994). The ecology of the Hong Kong limpets Cellana grata (Gould 1859) and Patelloida pygmaea (Dunker 1860): reproductive biology. *The Journal of Molluscan Studies*.](http://paperpile.com/b/SLRoeo/IKr5)

[Morton, B. & Morton, J. (1983). *The Sea Shore Ecology of Hong Kong*. Hong Kong University Press.](http://paperpile.com/b/SLRoeo/kcZG)

[Orton, J.H., Southward, A.J. & Dodd, J.M. (1956). Studies on the biology of limpets: II. The breeding of Patella vulgata L. in Britain. *Journal of the Marine Biological Association of the United Kingdom*, **35**, 149–176.](http://paperpile.com/b/SLRoeo/v6CC)

[Sin, T.M., Ang, H.P., Buurman, J., Lee, A.C., Leong, Y.L., Ooi, S.K., Steinberg, P. & Teo, S.L.-M. (2016). The urban marine environment of Singapore. *Regional Studies in Marine Science*, **8**, 331–339.](http://paperpile.com/b/SLRoeo/4byK)

[Underwood, A.J. (1974). The reproductive cycles and geographical distribution of some common eastern Australian prosobranchs (Mollusca: Gastropoda). *Marine and Freshwater Research*, **25**, 63–88.](http://paperpile.com/b/SLRoeo/h0rR)

[Underwood, A.J. & Keough, M.J. (2001). Supply-side ecology: the nature and consequences of variations in recruitment of intertidal organisms. *Marine Community Ecology. Sinauer Associates Inc, Sunderland*, 183–200.](http://paperpile.com/b/SLRoeo/SZWW)

[Wood, S.N. (2006). Generalized Additive Models: an introduction with R. CRC Press, Boca Raton, FL.](http://paperpile.com/b/SLRoeo/1rGL)
